# Predicting individual-specific cardiotoxicity responses induced by tyrosine kinase inhibitors

**DOI:** 10.3389/fphar.2023.1158222

**Published:** 2023-04-10

**Authors:** Jaehee V. Shim, Yuguang Xiong, Priyanka Dhanan, Rafael Dariolli, Evren U. Azeloglu, Bin Hu, Gomathi Jayaraman, Christoph Schaniel, Marc R. Birtwistle, Ravi Iyengar, Nicole C. Dubois, Eric A. Sobie

**Affiliations:** ^1^ Department of Pharmacological Sciences, Icahn School of Medicine at Mount Sinai, New York, NY, United States; ^2^ Institute for Systems Biomedicine, Icahn School of Medicine at Mount Sinai, New York, NY, United States; ^3^ Department of Cell, Developmental and Regenerative Biology, Icahn School of Medicine at Mount Sinai, New York, NY, United States; ^4^ Division of Nephrology, Department of Medicine, Icahn School of Medicine at Mount Sinai, New York, NY, United States

**Keywords:** mathematical model, cardiotoxicity, IPSC-CM cardiomyocytes, mRNASeq, mRNA sequencing, kinase inhibitor

## Abstract

**Introduction:** Tyrosine kinase inhibitor drugs (TKIs) are highly effective cancer drugs, yet many TKIs are associated with various forms of cardiotoxicity. The mechanisms underlying these drug-induced adverse events remain poorly understood. We studied mechanisms of TKI-induced cardiotoxicity by integrating several complementary approaches, including comprehensive transcriptomics, mechanistic mathematical modeling, and physiological assays in cultured human cardiac myocytes.

**Methods:** Induced pluripotent stem cells (iPSCs) from two healthy donors were differentiated into cardiac myocytes (iPSC-CMs), and cells were treated with a panel of 26 FDA-approved TKIs. Drug-induced changes in gene expression were quantified using mRNA-seq, changes in gene expression were integrated into a mechanistic mathematical model of electrophysiology and contraction, and simulation results were used to predict physiological outcomes.

**Results:** Experimental recordings of action potentials, intracellular calcium, and contraction in iPSC-CMs demonstrated that modeling predictions were accurate, with 81% of modeling predictions across the two cell lines confirmed experimentally. Surprisingly, simulations of how TKI-treated iPSC-CMs would respond to an additional arrhythmogenic insult, namely, hypokalemia, predicted dramatic differences between cell lines in how drugs affected arrhythmia susceptibility, and these predictions were confirmed experimentally. Computational analysis revealed that differences between cell lines in the upregulation or downregulation of particular ion channels could explain how TKI-treated cells responded differently to hypokalemia.

**Discussion:** Overall, the study identifies transcriptional mechanisms underlying cardiotoxicity caused by TKIs, and illustrates a novel approach for integrating transcriptomics with mechanistic mathematical models to generate experimentally testable, individual-specific predictions of adverse event risk.

## Introduction

Tyrosine kinase inhibitors (TKIs) have revolutionized the treatment of several types of cancer. In contrast to conventional chemotherapeutics, these drugs are targeted treatments designed to inhibit particular protein kinases that are abnormally active in specific cancers. TKIs, of which over 40 have been approved by the US Food and Drug Administration (Gharwan and Groninger, 2016), effectively treat cancers in many organs. For instance, imatinib has increased the survival rate of chronic myeloid leukemia patients by more than 90% (Yun et al., 2016), and trastuzumab has increased the survival of ERBB2^+^ breast cancer patients by 20% (Slamon et al., 2001). Unfortunately, many TKIs cause cardiotoxicity in small but significant numbers of cancer patients. Heart failure, ventricular arrhythmia, and hypertension, for example, have all been reported in response to treatment with TKIs (Force and Kolaja, 2011; Ewer and Ewer, 2015; Shim et al., 2017). Despite recent research efforts (Sharma et al., 2017; Wang et al., 2019), mechanisms underlying these cardiotoxicites remain poorly understood, and safety pharmacologists lack sensitive and selective preclinical assays to test new compounds for potential cardiotoxicity early in drug development.

Three major challenges inhibit both our understanding of TKI-induced cardiotoxicity and our ability to detect this adverse event in preclinical studies. One is the poor predictive value of animal models such as rats and rabbits (Ewart et al., 2014; Milani-Nejad and Janssen, 2014; Van Norman, 2019). A second challenge arises with *in vitro* cell culture assays, such as cardiomyocytes derived from human induced pluripotent stem cells (iPSC-CMs). This approach has gained considerable momentum recently, and several recent studies have obtained important insight into TKI-induced cardiotoxicity (Sharma et al., 2017; Wang et al., 2019). Studies in iPSC-CMs, however, still suffer from the lack of obvious cellular endpoints. Cell viability can easily be assessed after applying high concentrations of TKIs (Sharma et al., 2017), but this extreme perturbation is not representative of the more subtle changes that develop in patients’ hearts, generally over a time scale of weeks to months. A third challenge is the fact that, as with many adverse events, TKIs only cause cardiotoxicity in a relatively small percentage of patients. Thus, in addition to determining whether a new compound increases the aggregate risk of cardiac events, pharmacologists and clinicians would like to know which patients might be especially susceptible or resistant to cardiotoxicity. Answering this question may in the future allow TKIs to be administered more precisely, to patients who are likely to tolerate particular drugs.

We sought to determine whether integrated analysis approaches could successfully leverage molecular data to improve the use of *in vitro* experiments for prediction and understanding of TKI-induced cardiotoxicity. Specifically, we hypothesized that incorporating transcriptomic data into mechanistic mathematical models would generate experimentally-testable predictions and provide new insight. To test these ideas, iPSC-CM cell lines from two healthy donors were treated with 26 FDA-approved TKIs, and drug-induced changes in gene expression were quantified by bulk mRNAseq after 48 h. Based on changes in expression caused by TKIs, we developed a computational pipeline that allowed us to simulate, with a mechanistic model, how the drug-perturbed iPSC-CMs would respond to additional pathophysiological stimuli. We experimentally tested the modeling predictions and observed a strong correspondence in physiological outcomes between simulations and experiments. Somewhat surprisingly, we found consistent and repeatable differences in the susceptibility of specific iPSC-CM lines to particular drugs, and these could be explained by differences in drug-induced transcriptional changes. The results therefore demonstrate how mathematical models can be used as a mechanistic bridge between transcriptomic data and physiological outcomes, thereby improving our understanding of individual-specific susceptibility to cardiotoxicity.

## Materials and methods

### Study overview

Induced pluripotent stem cells (iPSCs) from two healthy female volunteers were differentiated into ventricular-like iPSC-CMs. These iPSCs were from a library generated in a previous study (Schaniel et al., 2021), which obtained skin fibroblasts from healthy volunteers and reprogrammed the cells into iPSCs. That study was approved by the Mount Sinai Institutional Review Board (protocol HS# 14–00530).

After differentiating the iPSCs into iPSC-CMs, cells were treated with TKIs at Day 30 after differentiation initiation, and comprehensive transcriptomic screening was performed using mRNAseq to quantify how 48 h treatment with 26 FDA-approved drugs influenced gene expression. Table 1 indicates the drugs used in the study and the concentration of each drug applied to the iPSC-CM cultures. Genes corresponding to parameters in a mechanistic mathematical model were extracted, fold changes compared with untreated cells were calculated, and simulations were performed to predict drug-induced changes in cellular action potentials, intracellular [Ca^2+^], contraction, and vulnerability to proarrhythmic insults. Simulation results were used to prioritize experimental tests, and physiological optical recordings were performed to test key predictions. Experimental methods describing cell generation and drug treatments are described in Supplemental Methods; approaches that were developed for this study, such as integration of mRNAseq data with mathematical models, application of secondary insults, and experimental prioritization, are described here.

**TABLE 1:** TKI treatment concentration and the solvent information. The stock solution of each TKI was diluted to the working concentration using the corresponding solvent. The appropriate amount of working concentration was then added to the media in the Petri dish containing iPSC-CMs to arrive to the final concentration.

Drug_ID	DrugName	Stock concentration (mM)	Working concentration	Final concentration	Solvent
**AFA**	Afatinib	10	50 uM	50 nM	DMSO
**AXI**	Axitinib	10	250 uM	200 nM	DMSO
**BEV**	Bevacizumab	0.168	0.5 mM	3uM	Water
**BOS**	Bosutinib	10	3 mM	100 nM	DMSO
**CAB**	Cabozantinib	10	1 mM	2 uM	DMSO
**CER**	Ceritinib	10	200 uM	1 uM	DMSO
**CRI**	Crizotinib	10	0.168 mM	0.25 uM	DMSO
**CTX**	Cetuximab	0.014	0.3 mM	1 uM	Water
**DAB**	Dabrafenib	10	100 uM	2.5 uM	DMSO
**DAS**	Dasatinib	10	2 mM	0.1 uM	DMSO
**ERL**	Erlotinib	12	3 mM	3 uM	DMSO
**GEF**	Gefitinib	10	30 mM	1 uM	DMSO
**IMA**	Imatinib	10	1 mM	5 uM	Water
**LAP**	Lapatinib	10	0.25 mM	2 uM	DMSO
**NIL**	Nilotinib	12	0.014 mM	3 uM	DMSO
**PAZ**	Pazopanib	10	125 uM	10 uM	DMSO
**PON**	Ponatinib	10	0.333 mM	100 nM	DMSO
**REG**	Regorafenib	10	2.5 mM	1 uM	DMSO
**RTX**	Rituximab	0.07	0.1 mM	3 uM	Water
**SOR**	Sorafenib	10	1 mM	1 uM	DMSO
**SUN**	Sunitinib	10	1 mM	1 uM	DMSO
**TOF**	Tofacitinib	10	10 mM	1 uM	DMSO
**TRA**	Trametinib	10	5 mM	100 nM	DMSO
**TRS**	Trastuzumab	3	1 mM	3 uM/10uM	Water
**VAN**	Vandetanib	10	10uM	333 nM	DMSO
**VEM**	Vemurafenib	10	1 mM	2uM	DMSO

### Differentiation of iPSCs into cardiomyocytes

Methods for differentiation of iPSC-CMs and assessment of cell purity, summarized briefly here, are described in detail in Supplementary Materials and relevant citations. In brief, iPSCs were differentiated using an established protocol (Yang et al., 2008; Kattman et al., 2011) that included generation of embryoid bodies and sequential activation and inhibition of WNT signaling. This protocol robustly produced CMs, as assessed by expression of 12 cardiac genes (e.g., cTNT and MLC2v), beat frequency, electrophysiology, and sarcomere formation. At day 20 EBs were dissociated and plated on plastic coverslips to form monolayers. Differentiation efficiency was then assessed by flow cytometry analysis for SIRPA, a cell surface marker that permits iPSC-CM identification and isolation (Dubois et al., 2011).

To improve purity for downstream assays, we used a metabolic selection protocol (Tohyama et al., 2013) that exploits the substrate flexibility of CMs compared with other cell types present in the cultures (primarily fibroblasts). Monolayers of cells were switched from glucose-containing media to lactate-containing media for 4 days, which increased iPSC-CM purity to roughly 95% after return to glucose media. See Supplementary Materials for details regarding media components and timing.

### Quantification of drug-induced changes in gene expression

The mRNAseq libraries were prepared using a 3′-Digital Gene Expression (DGE) method (Xiong et al., 2017). This method is a modified version of Single Cell RNA Barcoding and Sequencing method to handle extracted total RNA. The protocol starts with converting poly(A) mRNA to cDNA decorated with universal adapters, sample-specific barcodes and unique molecular identifiers (UMIs) using a template-switching reverse transcriptase. UMIs are unique to each transcript from which cDNA is generated. The cDNAs from multiple samples are then pooled and amplified for 10 PCR cycles. The pooled amplicons are fragmented using a modified transposon-based method, then prepared for multiplexed sequencing. Sequencing was performed using a HiSeq 2,500 with the customized sequencing setting; paired end with 26 base pairs was sequenced in the first read and the 46 base pairs were sequenced in the second read. Transcriptomic sequences were aligned with STAR (Dobin et al., 2013) and normalized with edgeR (Robinson et al., 2010). Details of data processing and links to SOPs are provided in the Supplementary Materials.

### Transcriptomics-based EC coupling simulations

The mathematical model utilized in this study was constructed by integrating a model of human iPSC-CM electrophysiology and Ca^2+^ handling (Paci et al., 2013) with a cardiac myofilament model (Rice et al., 2008) such that the combined model simulated each step from electrical excitation through contraction (Supplementary Figure S1). Parameters controlling contraction were modified to compensate for lower systolic [Ca^2+^] levels in iPSC-CMs compared with adult ventricular myocytes (see Supplementary Methods).

To simulate how TKIs may influence iPSC-CM physiology, we extracted from mRNA-seq datasets the genes that correspond to parameters in the mathematical model (Table 2). The underlying premise of our approach was that even if mRNA levels cannot generally be mapped directly to model parameters, drug-induced changes in ion transport pathways can be represented by calculating mRNA fold changes in the drug-treated compared with the untreated state. For the electrophysiology part of the model (Paci et al., 2013), measurements of ion channel gene expression were used to scale conductance parameters in the model. First, we computed the weighted sum of channel genes for each parameter, then calculated the fold change to determine how to modify each parameter in drug-treated compared with control iPSC-CMs. These values were then used as scale factors to modify each parameter by multiplication.

$$ScaleFactor_{TKI}=\frac{\Sigma \left(Y_{TKI}\right)}{\Sigma \left(Y_{CTRL}\right)},Y=gene\;expression\;for\;relevant\;channel$$

$${G_{ion\_new}=G}_{ion\_default}\times {ScaleFactor}_{TKI}$$

**TABLE 2:** Genes used to compute scaling factors for the 15 model parameters.

Parameters	Gene ID
G_Na_	SCN5A
G_CaL_	CACNA1C * all voltage gated calcium channel *
	CACNA1S,CACNA1D,CACNA1B,CACNA1I,CACNA1G,CACNA1H,CACNA1A,CACNA1E,CACNA1F,CACNA1C,CACNA2D1
G_RYR_	RYR2
G_to_	KCND2, KCND3, KCNA4, KCNA7
G_K**s**_	KCNQ1, KCNE1
G_Kr_	KCNH2
G_K1_	KCNJ2, KCNJ12
P_NaK_	ATP1A1
I_up_	ATP2A2
G_pCa_	ATP2B4
G_f_	HCN2, HCN4
K_NaCa_	SLC8A1
Trop_Conc	TNNC1
Myosin	MYH6, MYH7
Actin	ACTC1

Each new conductance was used to compute the relevant ionic current, with model equations otherwise unmodified.

Contractile gene integration was performed using troponin C, actin, and myosin heavy chain (MHC) expression. Troponin C gene expression was integrated in an identical manner to ion channels; integration of actin and MHC involved more steps, as described in Supplemental Methods.

### Calculation of arrhythmia risk after simulations of secondary insults

To predict how drug-induced changes in gene expression would interact with environmental factors to influence arrhythmia risk, we simulated the responses of drug-treated iPSC-CMs to three secondary insults: 1) an increase in L-type Ca^2+^ current, 2) block of delayed rectifier K^+^ current (I_Kr_), and 3) hypokalemia. For hypokalemia simulations, we initially verified that K^+^ currents in the Paci et al. model of iPSC-CM electrophysiology (Paci et al., 2013) responded to changes in extracellular [K^+^] in a similar manner to those seen in adult myocytes (Trenor et al., 2018). Having established this, we then performed secondary insult simulations by scaling relevant model parameters at 10 different values representing mild to severe changes. Simulations of 1 Hz pacing were run for 120 s, and the last 3 seconds of CaT and AP waveforms were analyzed for morphology changes and the potential appearance of arrhythmic behavior. Either early afterdepolarizations (EADs) or failure to repolarize were considered arrhythmic events, and the minimal perturbation level that produced arrhythmic behavior was considered the threshold. For each TKI, we synthesized results from all 3 secondary insults to compute an Arrhythmic Index (AI), as described in Supplementary Methods.

For simulations to determine mechanisms underlying arrhythmic susceptibility, we examined in 8 major ionic currents (I_K1_, I_to_, I_Kr_, I_Ks_, I_CaL_, I_NaK_, I_Na_, I_NCX_). Over a single action potential, we integrated the total charge through each current and calculated how drug -induced gene expression changes affected the total amount of charge through that current, compared with untreated cells. At each level of secondary insult, this was expressed as ΔQ, the change in integrated current in the drug-treated compared with the untreated state. For I_NCX_, which reverses during the action potential, we computed ΔQ for outward and inward currents separately (Supplementary Figure S4). A negative ΔQ, which can arise from either an increase in inward (negative) current or a decrease in outward (positive) current, inhibits repolarization and is considered proarrhythmic.

### Optical recordings of action potentials, intracellular [Ca^2+^], and membrane movement

To test predictions of mechanistic simulations, action potentials and membrane movement were optically recorded in iPSC-CMs loaded with the voltage sensitive dye FluoVolt, and intracellular [Ca^2+^] was recorded in separate experiments in cells loaded with the Ca^2+^ indicator fluo3. The Supplemental Methods contain details on cell plating, dye loading, solutions, and line scan recordings made with a confocal microscope.

Recordings were first made during steady-state 1 Hz pacing with 5.4 mM extracellular [K^+^]. Hypokalemia was then applied by progressively lowering extracellular [K^+^] to 4.1, then 2.9, then 2.5 mM. At each level of [K^+^], recordings were made after 1 Hz pacing for 10 min. On each experimental cover slip, recordings were made from 5–10 locations at each level of extracellular [K^+^]. Both the total number of recordings (n) and the total number of cover slips (N) are reported for each condition.

Metrics that were derived from FluoVolt membrane potential measurements included action potential duration, action potential triangulation, and membrane movement. Metrics computed from fluo3 measurements were Ca^2+^ transient Decay Tau, Ca^2+^ transient triangulation, and Ca^2+^ transient Area Under the Curve (AUC). Membrane movement, an indirect measure of iPSC-CM contraction, was quantified by deflections in line-scan fluorescence recordings, as illustrated in Supplementary Figure S3. Details are provided in Supplementary Methods and illustrated schematically in Supplementary Figure S3.

### Statistical analysis

All data presented with error bars are means ± σ and statistical significance is reported at three levels: *p < 0.05, **p < 0.01 and ***p < 0.001. One tailed, two sample, unpaired *t*-test was performed for evaluating potential differences between drug-treated and vehicle-treated groups. The MATLAB function *ttest2* was used to perform the *t*-test, and unequal variance was assumed for two sample comparisons. For studying interactions between the secondary insult and TKI treatments, two-way ANOVA was performed using MATLAB statistics function *anovan* with ‘model’ argument set to 2.

## Results

### Differentiation of iPSCs yields pure populations of cardiomyocytes

**FIGURE 1:**
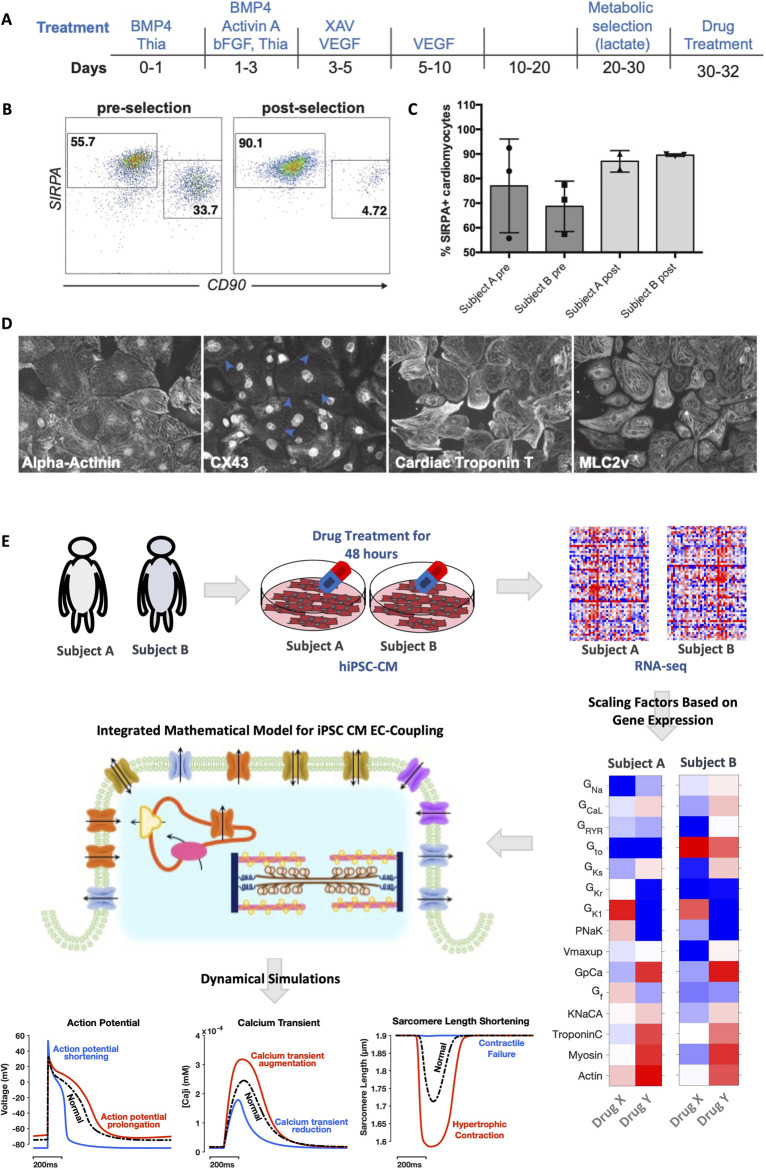
Preparation of purified iPSC-CMs and computational pipeline for simulation analysis. **(A)** Schematic of differentiation and metabolic selection of human pluripotent stem cells. **(B)** Flow cytometry analysis for SIRPA (cardiomyocyte marker) and CD90 (fibroblast marker) at day 20 (before lactate selection) and day 30 (after lactate selection). **(C)** Quantification of SIRPA^+^ cells before and after lactate selection in the two cell lines, based on n = 3 differentiations in each cell line. **(D)** IF analysis of iPSC-CMs after lactate selection at day 30, Cells were stained with antibodies against α-actinin, Connexin 43 (CX43), cardiac Troponin T and MLC2v. **(E)** Study workflow illustrates how iPSCs derived from two healthy human volunteers were differentiated into cardiac myocytes, then treated with 26 FDA-approved tyrosine kinase inhibitor drugs. Gene expression was quantified in each cell line with mRNA-seq, and genes relevant to iPSC-CM excitation-contraction coupling were extracted and converted into fold changes, indicating relative changes resulting from drug treatment. These fold changes corresponded to alterations in mathematical model parameters, specific to each cell line, and simulations predicted changes in action potentials, intracellular Ca^2+^ transients, and sarcomere shortening caused by drugs.

Human iPSCs were generated by reprogramming skin fibroblasts from two healthy donors. The cells were then differentiated into cardiomyocytes (CMs) as illustrated schematically in Figure 1A (see Supplemental Methods for details). A lactate selection protocol (Tohyama et al., 2013), applied between days 20 and 30, increased the percentage of CMs in the culture, as assessed by flow cytometry for the CM membrane marker SIRPA (Dubois et al., 2011) (Figure 1B). Across the two iPSC lines, lactate selection increased the percentage of SIRPA^+^ cells from 60%–75% before lactate selection to ∼90% after lactate selection (Figure 1C). The differentiation protocol yielded iPSC-CMs that beat spontaneously and expressed canonical cardiac markers, including α-actinin, Connexin-43, Troponin T, and Myosin Light Chain 2v (Figure 1D). Purified iPSC-CM cultures were used for all downstream experiments, including treatment with TKIs, quantification of gene expression using mRNA-seq, and optical measurements of action potentials (APs) and intracellular Ca^2+^ transients (CaTs).

### Integration of gene expression data with mathematical modeling generates individual-specific and experimentally-testable predictions of TKI-induced effects

Figure 1E illustrates the overall strategy of the study, described in more detail in Methods and Supplementary Materials. After iPSC-CMs were treated with 26 FDA-approved TKIs for 48 h, mRNAseq was performed to quantify drug-induced changes in gene expression. From these genome-wide transcriptomic measurements, a subset of genes was extracted that corresponds with parameters in an integrated model of iPSC-CM electrophysiology and contraction (Rice et al., 2008; Paci et al., 2013) (see Table 2 and Methods). From these selected genes, fold changes were calculated to quantify, for the two cell lines, how much each TKI either increased or decreased each model parameter compared with vehicle-treated control iPSC-CMs. Simulations were then performed to predict how these TKI-induced changes in gene expression affected APs, CaTs, and sarcomere length shortening (Figure 1E, bottom). Differences between the two cell lines in TKI-induced gene expression produced differences in physiological predictions.

### Simulations predict individual-specific alterations to AP, CaT, and SL shortening waveforms

**FIGURE 2:**
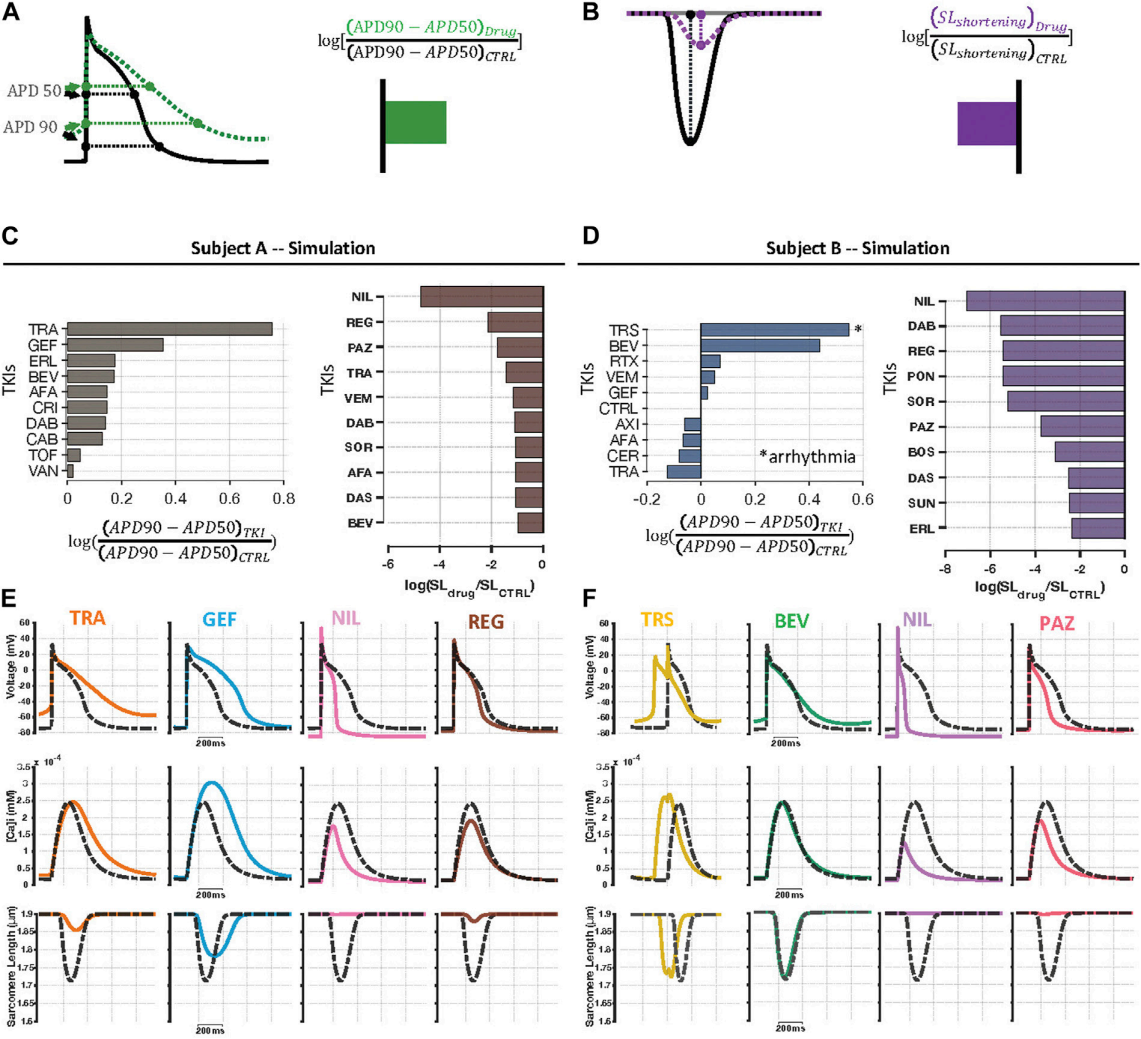
Individual specific predictions of physiological alterations caused by TKIs. **(A)** To examine TKI-induced effects on electrophysiology, we computed triangulation of AP waveforms from the simulation output for each drug. In the diagram, the green curve, representing drug-induced changes, shows a more triangular waveform than the black curve, representing the baseline model, which indicates an increase in triangulation. **(B)** TKI-induced contractile dysfunction was evaluated using sarcomere length shortening simulation results. Because the drug-treated purple curve exhibits reduced shortening compared with the black (control) curve, this change is summarized as a decrease in contraction strength. **(C, D)** Individual specific, top 10 rankings for log-transformed AP triangulation and contractile failure metrics. Amongst the top 10 most highly-ranked TKIs, there was higher level of correlation between subject A and B in contractile dysfunction (Spearman’s rank correlation, ρ = 0.64, *p* = 0.054) than AP triangulation rankings (ρ = -0.16, *p* = 0.65). **(E, F)** Example AP, CaT, and SL shortening simulation results in the two cell lines showing examples of drug-induced AP triangulation and contractile failure. Dashed black curves represent untreated cells, and colored lines represent predictions for, from left to right, trametinib, gefitinib, nilotinib, and regorafenib in Cell Line A, and trastuzumab, bevacizumab, nilotinib, and pazopanib in Cell Line B.

Simulations performed with the integrated iPSC-CM mathematical model (Rice et al., 2008; Paci et al., 2013) predicted how measured TKI-induced gene expression changes in each cell line modified AP and SL shortening waveforms. For each drug and each cell line, we examined drug-induced changes in simulated time courses (compared with untreated cells) and ranked predicted alterations in AP triangulation (a known indicator of proarrhythmia) and sarcomere shortening (Figures 2A,B). The top ten rankings of AP triangulation revealed little overlap between the cell lines from the two individuals (Figure 2Ci versus Figure 2Di). In contrast, the rankings for the fold reduction of SL shortening appeared more similar between the two cell lines (Figure 2Cii versus Figure 2Dii). These impressions were confirmed by calculations of Spearman’s rank correlation (*ρ* = -0.16 for triangulation, and *ρ* = 0.64 for SL shortening). Example simulations from drugs that caused triangulation or contractile failure are shown in Figures 2E,F. In subject A (Figure 2E), trametinib and gefitinib are shown to cause depolarization of the resting membrane potential, AP triangulation (top), moderate triangulation of Ca^2+^ transients (middle), and moderate reductions in contraction (bottom). In subject B, in contrast, trastuzumab and bevacizumab were predicted to cause AP triangulation (Figure 2F). Interestingly, in the trastuzumab simulations, spontaneous depolarization preceded the electrical stimulus, which is classified as a form of arrhythmia when cells are paced at 1 Hz (Figure 2F). In terms of drug effects on contraction, nilotinib and regorafenib are predicted to virtually eliminate contraction in cells from subject A, whereas nilotinib and pazopanib are predicted to do the same in subject B. Interestingly, contractile failure can be accompanied by a severe reduction in Ca^2+^ transient amplitude (e.g., nilotinib in either subject), or little change in Ca^2+^ transient amplitude (regorafenib in subject A and pazopanib in subject B). These simulations therefore generate predictions that can be both tested experimentally and examined in mechanistic detail.

### Cellular physiology experiments confirm individual-specific drug responses

**FIGURE 3:**
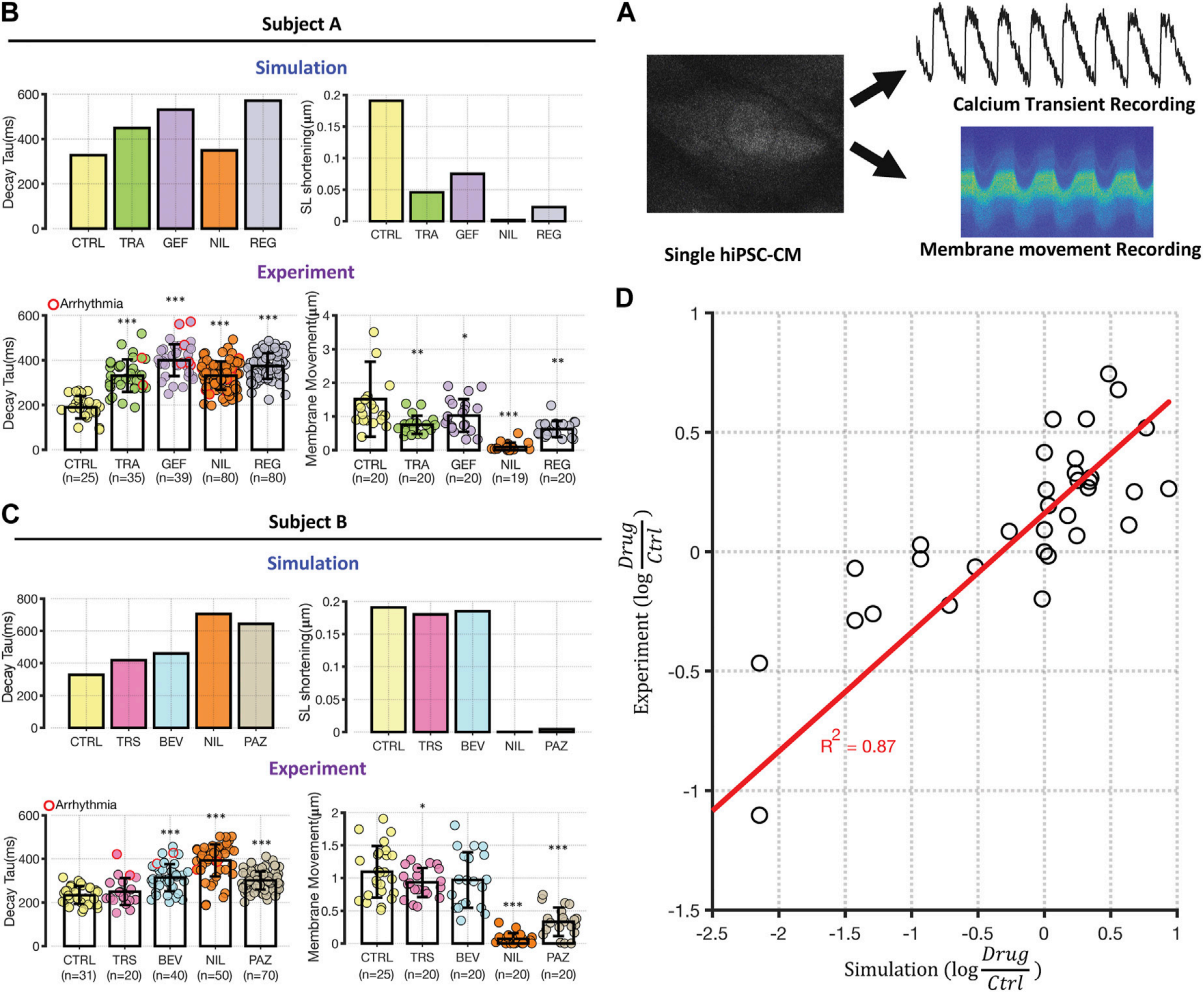
Experimental testing of transcriptomic based simulation predictions. **(A)** Schematic illustrating that iPSC-CMs were loaded with either fluo3 or FluoVolt for CaT or membrane movement recordings, respectively. Metrics were extracted from fluorescence time course measurements for comparison with simulation results. **(B, C)** Comparison between simulations of Decay Tau (left bar graphs) and SL shortening (right bar graphs) in the two cell lines. Simulation results for control conditions and four drugs in each cell line are shown above experimental results under the same conditions. Results from Cell Lines A and B are displayed in panels **(B)** and **(C)**, respectively. Error bars indicate standard deviation, and asterisks indicate significantly different from control, based on two sample, unpaired *t*-test (*p < 0.05, **p < 0.01 and ***p < 0.001). The number of experimental samples in each group is provided beneath the experimental bar graphs. **(D)** Direct comparison of modeling predictions (abscissa) with experimental data (ordinate), with each expressed as the logarithm of the change in drug-treated relative to vehicle-treated iPSC-CMs. Each dot represents a change in a time course metric (Decay Tau, CaT triangulation, CaT AUC, and SL shortening) caused by changes in gene expression induced by a particular drugs. Results from Cell Lines A and B are grouped together, and the calculated R^2^ of 0.87 includes 3 data points that induce extreme changes to metrics and are not visible on this scale.

To test the modeling predictions, we loaded iPSC-CMs with fluorescent dyes and used confocal microscopy to record APs, intracellular [Ca^2+^], and membrane movement (Figure 3A; see Methods for details). We tested 4 TKIs in each cell line and used simulation results to prioritize experimental tests by selecting drugs that were predicted to: 1) cause AP triangulation, impaired contraction, or both, and 2) when possible, cause divergent effects between the two cell lines. For arrhythmia related metrics, we evaluated the time constant of [Ca^2+^] decay (Decay Tau) and CaT triangulation (CaD_90_/CaD_50_). These metrics are associated, respectively, with the reduced SERCA activity that occurs in heart failure (Kho et al., 2012), and AP triangulation, an established proarrhythmic index (Shah and Hondeghem, 2005). To evaluate effects on myocyte contraction, we examined the area under the CaT waveform (CaT AUC), a rough approximation of the amount Ca^2+^ available to produce contraction, and cell shortening, defined as sarcomere shortening in simulations and membrane movement in experiments.

Modeling predictions and experimental measurements of Decay Tau and cell shortening are shown in Figures 3B,C; the remaining metrics are shown in Supplementary Figure S7. To validate the initial impression that most predictions were validated, we quantified prediction accuracy in two ways. First, we classified each simulation as predicting an increase (>20%), a decrease (<20%), or no change, for each combination of metric, drug, and cell line. Experimental recordings were then defined as either consistent with or inconsistent with predictions, based on whether statistically significant changes in the same direction were produced. Combining both proarrhythmia and contraction metrics, we observed confirmation of 75% of predictions (12/16) in cell line A and 87.5% of predictions (14/16) in cell line B. Second, we calculated the TKI-induced change in each metric, relative to vehicle-treated control cells, in both simulations and experiments, and observed a strong correlation between model predictions and data (R^2^ = 0.87, Figure 3D). Overall, the consistency of physiological recordings with model predictions bolsters the validity and robustness of our computational approach.

### Modeling provides insights into mechanisms underlying contractile failure

A striking finding in Figure 3 was the prediction that certain drugs (regorafenib in Cell Line A, pazopinib in Cell Line B, nilotinib in both cell lines) caused the virtual elimination of cellular contraction, predictions that were confirmed in experimental measurements of membrane movement. Because myocyte contraction is initiated by the binding of Ca^2+^ to cardiac troponin-C, which enables actin and myosin to interact, elimination of contraction could occur through a severe reduction in intracellular [Ca^2+^], or through reduced expression of genes encoding contractile proteins (e.g., *MYH7*, *ACTC1*, *TNNC1*). Some TKIs, such as nilotinib, were predicted to produce contractile failure through a combination of reduced CaT AUC and downregulation of contractile genes. In contrast, simulations predicted much smaller changes in CaT AUC resulting from regorafinib in Cell Line A or pazopinib in Cell Line B, and experiments failed to observe significant changes in this metric. In these cases, contractile failure resulted from dramatic downregulation of genes such as *MYH7* and *ACTC1*.

### Secondary insults can potentiate TKI-induced arrhythmogenicity

**FIGURE 4:**
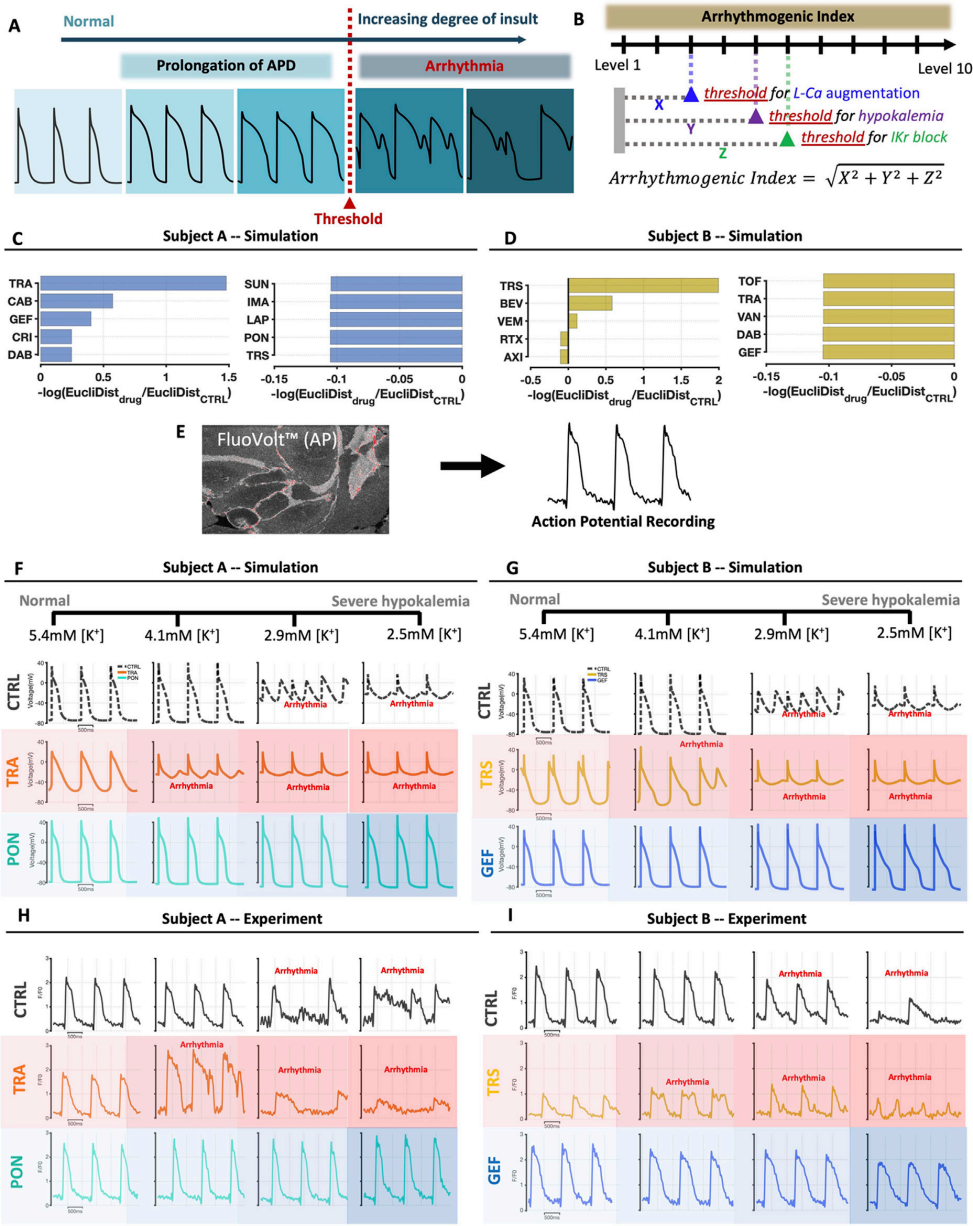
Simulations and recordings show that the response of iPSC-CMs to arrhythmogenic secondary insults is drug-specific and cell line-specific. **(A)** Schematic illustrating the calculation of iPSC-CM susceptibility to secondary insults. In simulations, the degree of insult is progressively increased, and the level at which arrhythmic dynamics such as early afterdepolarizations are seen is taken as the threshold. **(B)** Arrhythmic index (AI), for each drug and in each cell line, is calculated as a weighted average of threshold values for three insults applied, with data transformations applied such that positive AI represents increased susceptibility, and negative AI represents reduced susceptibility. **(C, D)** Bar graphs show the 5 drugs with the largest and smallest values of AI, in Cell Lines A and B respectively. Note that AI was computed for all 26 drugs tested in the study; results from 16 simulations in each cell line are not shown. **(E)** Schematic illustrating that APs were recorded in iPSC-CMs loaded with the fluorescent membrane potential indictor FluoVolt. **(F, G)** Simulated AP traces in Cell Lines A and B, respectively, showing results in untreated cells, in cells treated with a TKI predicted to increase susceptibility, and in cells treated with a TKI predicted to decrease susceptibility. Results are shown at four different levels of hypokalemia, ranging from normal (5.4 mM) to severe hypokalemia (2.5 mM). **(H, I)** Exemplar AP recordings in Cell Lines A and B, respectively, showing results in untreated cells, in cells treated with a TKI predicted to increase susceptibility, and in cells treated with a TKI predicted to decrease susceptibility. Results are shown at four different levels of hypokalemia, ranging from normal (5.4 mM) to severe hypokalemia (2.5 mM).

An interesting finding from the simulations was that none of the TKIs, with the exception of trastuzumab in Cell Line B, was predicted to induce cellular arrhythmias. However, several TKIs were predicted to cause AP prolongation and triangulation, both of which are associated with higher arrhythmic risk (Shah and Hondeghem, 2005). Based on these results, we hypothesized that some TKIs might alter cell state such that myocytes become more susceptible to secondary insults. To test this idea, we performed simulations in which potentially-arrhythmogenic insults were applied to iPSC-CMs whose physiology had been altered by TKI-induced changes in gene expression. Specifically, we predicted the response of TKI-treated iPSC-CMs to three arrhythmogenic perturbations: 1) hypokalemia, i.e., a decrease in extracellular [K^+^]; 2) an increase in the magnitude of L-type Ca^2+^ current; and 3) block of the rapid delayed rectifier current, I_Kr_. In each case, we progressively increased the magnitude of the insult and documented the level that produced arrhythmic dynamics (Figure 4A). To synthesize results, we computed a risk score, arrhythmogenic index (AI), for each drug in each cell line, based on whether TKI-induced changes in gene expression made cells more or less susceptible to simulated proarrhythmic insults (see Materials and Methods).

Simulation results, shown in Figure 4C for Cell line A and Figure 4D for Cell Line B, showed several interesting features. First, TKI-induced changes in gene expression could either increase susceptibility (left bar plots), or decrease susceptibility and protect cells from arrhythmia (right plots). Second, we observed only moderate correspondence between the susceptibility rankings and the AP triangulation rankings shown in Figure 2. Third, and most surprisingly, dramatic differences were seen between the two cell lines in the drugs predicted to increase (or decrease) susceptibility. For instance, trametinib was predicted to be the most dangerous drug in Cell Line A but one of the most protective drugs in Cell Line B. Conversely, trastuzumab was predicted to decrease susceptibility in Cell Line A but increase susceptibility in Cell Line B. These simulations therefore support a “two-hit” hypothesis that myocyte dysfunction may be produced through a combination of TKI treatment and secondary perturbations. Moreover, these results provide individual-specific, experimentally-testable predictions.

### Action potential and intracellular [Ca^2+^] recordings validate the two-hit hypothesis with hypokalemia as the secondary insult

To test the surprising predictions that particular TKIs could either increase or decrease susceptibility to arrhythmogenic insults, depending on cell line, we recorded APs and intracellular [Ca^2+^] while subjecting iPSC-CMs to hypokalemia, a condition that can lead to AP prolongation and early afterdepolarizations (Weiss et al., 2017). Experiments were performed with two TKIs predicted to increase susceptibility, and two predicted to decrease susceptibility, in each cell line. When possible, we interrogated the individual-specific predictions by choosing TKIs whose predictions diverged between the cell lines. The protocol consisted of four levels of extracellular [K^+^] that span a range from normal [K^+^] to moderate hypokalemia: 5.4 mM (normal), 4.1 mM, 2.9 mM, and 2.5 mM. Simulation time courses (top) and exemplar AP recordings (bottom) are shown in Figures 4F–I, with additional results shown in Supplementary Figures S9, S10. Experimental results were cell line-specific and in agreement with modeling predictions. In the example shown, trametinib produced arrhythmic dynamics at 4.1 mM [K^+^] in Cell Line A, consistent with the increased susceptibility predicted by simulations, whereas normal dynamics were observed at all levels of [K^+^] in Cell Line B, consistent with the predicted protective effect.

**FIGURE 5:**
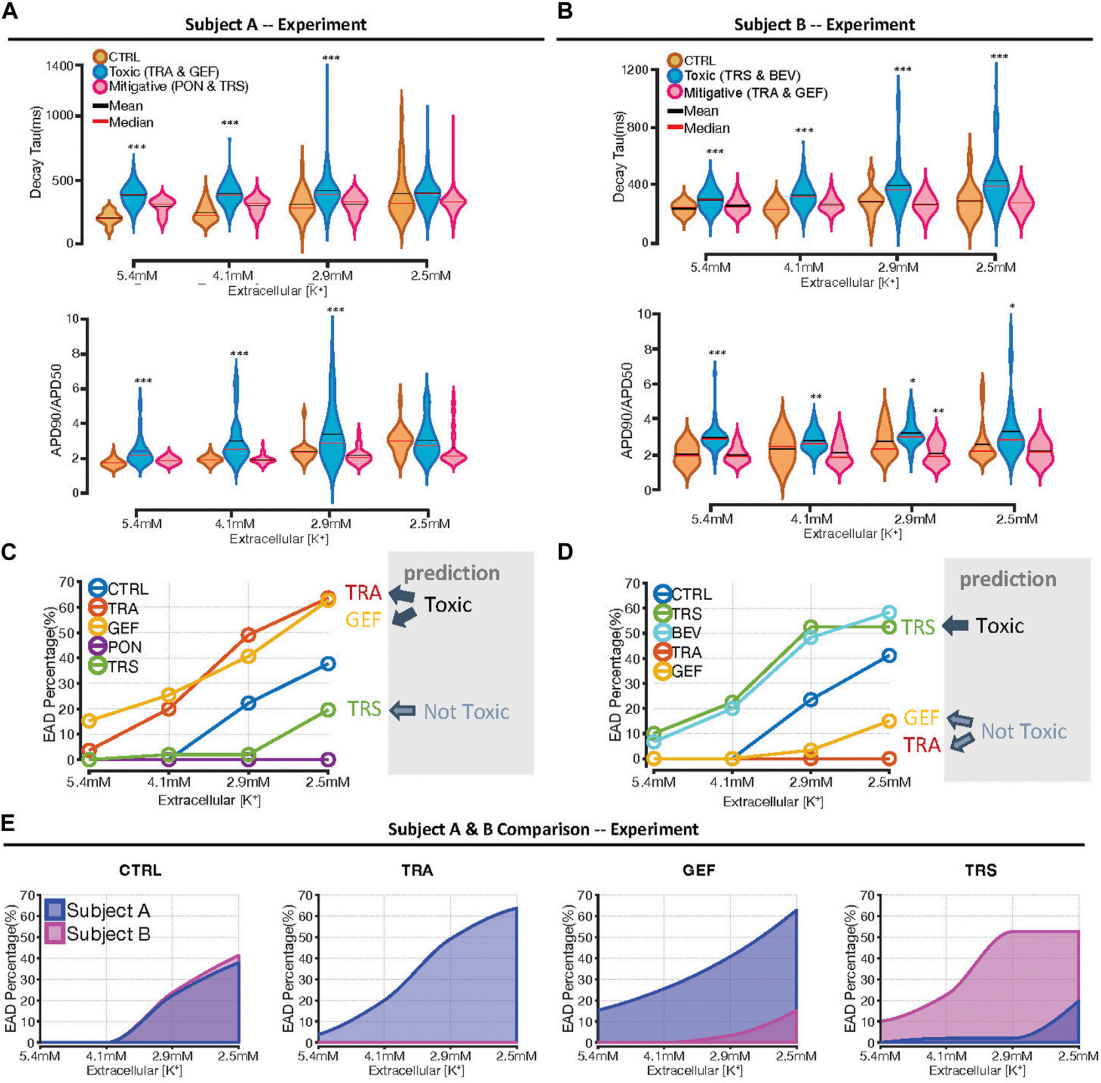
Summary data indicating differential response to hypokalemia between cell lines. **(A, B)** Violin plots indicating the distributions of Decay Tau (top plots), and AP triangulation (bottom plots), as a function of extracellular [K^+^], in Cell Lines A and B, respectively. To facilitate statistical comparisons, drug treatments are grouped as either “toxic” drugs or “mitigative” drugs predicted to increase or decrease susceptibility, respectively. Asterisks indicate conditions statistically different from vehicle-treated control cells at the same level of extracellular [K^+^], using a two sample, unpaired *t*-test (*p < 0.05, **p < 0.01 and ***p < 0.001). **(C, D)** Percentage of samples exhibiting arrhythmic dynamics, as a function of extracellular [K^+^], in Cell Lines A and B, respectively. Drugs predicted to be toxic, namely, trametinib and gefitinib in Cell Line A, and trastuzumab and bevacizumab in Cell Line B, exhibited an increase in arrhythmia percentage at all levels of extracellular [K^+^]. **(E)** Comparison of arrhythmia percentage between cell lines, as a function of [K^+^], under different conditions. The numbers of cells and cover slips under each condition are provided in Supplementary Table S1.

Summary data, shown in Figure 5, consist of CaT Decay Tau, AP triangulation, and the percentage of cells exhibiting arrhythmic dynamics at each level of [K^+^]. Consistent with expectations, reductions in extracellular [K^+^] led to increases in Decay Tau and AP triangulation in all groups. These changes, however, were larger in cells treated with the drugs predicted to increase susceptibility, whereas cells treated with drugs in the protective group showed values close to vehicle-treated control cells (Figures 5A,B). Two-way ANOVA reported significant interaction between TKI treatment and hypokalemia, indicating that the TKI-induced increase of Decay Tau and AP triangulation can be augmented by hypokalemia. Figures 5C,D, plotting arrhythmia percentages from all cells tested, confirm that trametinib and gefitinib increased susceptibility to hypokalemia in Cell Line A, whereas trastuzumab and bevacizumab increased susceptibility in Cell Line B. Figure 5E replots arrhythmia percentages for individual conditions to highlight differences between cell lines. In vehicle-treated control cells, arrhythmic dynamics were never observed at 5.4 or 4.1 mM [K^+^], then seen in 22%–26% of samples in either cell line at 2.9 mM [K^+^], and 36%–41% of samples at 2.5 mM [K^+^]. For the 3 drugs that were tested in both cell lines–trametinib, gefitinib, and trastuzumab–we observed a markedly higher percentage of arrhythmic dynamics in one cell line but a decrease in the other cell line, consistent with the modeling predictions (Figures 4C,D).

### Simulations reveal that drugs may increase arrhythmia susceptibility through downregulation of repolarizing ionic currents

**FIGURE 6:**
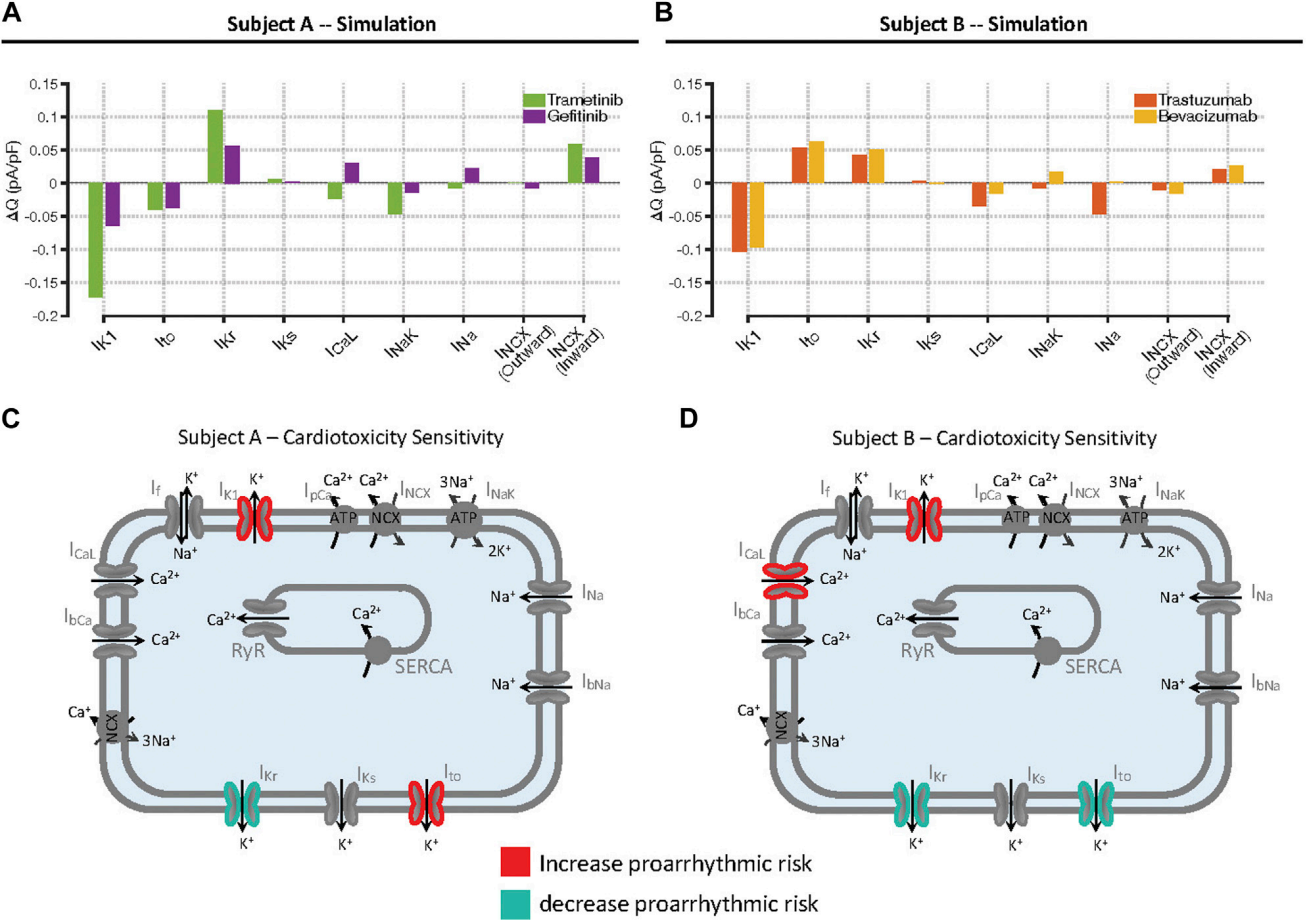
Mechanisms underlying increased arrhythmia susceptibility caused by individual drugs in the two cell lines. **(A, B)** Calculations of changes in total charge, ΔQ, passing through individual ionic currents, in Cell Lines A and B, respectively. Simulations were performed at the level of extracellular [K^+^] immediately before arrhythmias occurred in drug-treated cells, and the integral of each current during the action potential was computed. Bars represent the difference in integrated current, or charge (Q), between drug-treated and untreated cells. **(C, D)** Summary of changes in ionic currents that accounted for increased susceptibility in the two cell lines. Each panel shows the changes in ionic currents that act to increase arrhythmia risk in red, contrasted with the changes that act to decrease arrhythmia risk in blue.

The results shown above, while demonstrating differences in how individual drugs influence arrhythmia susceptibility between cell lines, do not fully reveal the underlying mechanisms, a question that can be explored through additional model simulations. To address potential mechanisms, we simulated action potentials and ionic currents in iPSC-CMs treated with drugs that increased susceptibility: trametinib and gefitinib in Cell Line A, and trastuzumab and bevacizumab in Cell Line B. At the level of extracellular [K^+^] that immediately preceded arrhythmia, we computed the integral of each ionic current during the AP and compared this with the integrated current in untreated cells, thereby calculating the difference in charge, or ΔQ, through each current. Plots of ΔQ for the model’s ionic currents reveals changes in ionic currents that are potentially responsible for increased susceptibility to hypokalemia in the two cell lines (Figures 6A,B). For instance, in Cell Line A both drugs produce an increase in rapid delayed rectifier I_Kr_ and a reduction in inward current through the Na^+^-Ca^2+^ exchanger, I_NCX_. These alterations produce positive ΔQ, which would protect cells from arrhythmia. These changes are offset, however, by reductions in transient outward current I_to_ and inward rectifier current I_K1_, alterations that increase susceptibility through negative ΔQ. Drug-induced changes to ionic currents in Cell Line B are somewhat similar in that both toxic drugs induce negative ΔQ though I_K1_ and a protective positive ΔQ through I_Kr_. In Cell Line B, however, increased I_to_ is potentially protective (positive ΔQ) whereas negative ΔQ though L-type Ca^2+^ current I_CaL_. may contribute to increased arrhythmia susceptibility. These changes, summarized schematically in Figure 6C, illustrate how mechanistic models can generate quantitative hypotheses for differences in susceptibility between drugs and between individuals, insights that cannot generally be obtained through the analysis of transcriptomic data alone. Simulations performed with additional drugs, including those that are protective, and illustrations of ΔQ calculations, are provided in Supplementary Figures S12, S13.

## Discussion

### Evidence supporting a new approach for addressing TKI-induced cardiotoxicity

The mechanisms underlying cardiotoxicity in some patients treated with TKIs remain unclear, and the elucidation of these mechanisms is a major research focus in cardio-oncology. Although the observed rates of adverse events are relatively low, a literature review conducted in 2017 found that 26 out of then 30 FDA-approved TKIs list some form of serious cardiotoxic effect in their black box warnings (Shim et al., 2017). Several issues, however, have inhibited our understanding of underlying cardiotoxic mechanisms. One is that animal models are only partially predictive of adverse events observed in humans (Ewart et al., 2014; Milani-Nejad and Janssen, 2014; Van Norman, 2019). A second complication is that TKI-induced adverse events develop over a time scale of weeks to months and are thought to involve drug-induced cardiac remodeling. The generally limited time scale of *in vitro* experiments, such as those performed with iPSC-CMs, calls into question their direct clinical utility. To attempt to overcome these limitations, we developed an integrative approach that combines the broad coverage provided by transcriptomic measurements in human iPSC-CMs with the mechanistic insights gained from dynamical modeling and physiological experiments. Our underlying premise is that 48 h drug treatments at clinically-relevant concentrations do not cause overt toxicity, but can produce changes in cell state that prime cells for pathophysiological responses to additional stimuli. The results that we obtained, through experimental tests of simulation predictions, corroborate this “two-hit” hypothesis and support our approach to TKI-induced cardiotoxicity as a strategy for screening and improved understanding.

One of our surprising simulation results was that predictions were specific to the cell lines obtained from different individuals. Whether we examined changes in AP shape due to TKI-induced changes in gene expression (Figure 2) or the response of iPSC-CMs to additional insults such as hypokalemia (Figure 4), we observed pronounced differences between cell lines in the rankings of drugs that were predicted to cause substantial effects. These differences informed our choices of which predictions to test in physiological experiments, allowing us to select drugs that were predicted to cause divergent outcomes between the cell lines. The experimental confirmation of these substantial differences between cell lines is illustrated most clearly in Figure 5E, which shows results from 3 drugs that increase susceptibility to hypokalemia in one cell line while decreasing susceptibility in the other cell line. Individual drugs could either cause upregulation of potentially protective currents, such as I_to_, or an arrhythmogenic downregulation of currents such as I_K1_, thereby providing mechanistic grounding for the differences between cell lines (Figure 6 and Supplementary Figures S12, S13). Currents that have been shown to be influenced by certain TKIs, such as the funny current I_f_ (Wu and Cohen, 1997), were also analyzed, but drug-induced changes were smaller than with the currents mentioned (Supplementary Figures S12, S13). Overall, the strong correspondence that we observed between modeling predictions (Figures 2, 4) and experimental results (Figures 3, 5) provides confidence that changes in gene expression can be combined with mechanistic models to produce individual-specific, experimentally-testable predictions. At one level, the cell line-specific results confirm what we already know intuitively; responses to drugs can be idiosyncratic (Vogenberg et al., 2010). More broadly, however, the research suggests how the strategy can be extended to gain insight into the small percentage of patients who may be especially susceptible to adverse events caused by a particular drug.

### Integration of omics data with mechanistic mathematical models

Statistical analyses of large scale Omics data on one hand, and mechanistic mathematical models on the other hand, are often considered the two main pillars of systems biology (Sobie et al., 2011; Janes and Lauffenburger, 2013). Our study illustrates a strategy to integrate these fundamentally different approaches in a manner that allows for experimental tests and mechanistic insight. Although it makes intuitive sense that changes in cell state captured by an Omics assay can inform the conditions for model simulations (Iyengar et al., 2012), the best methods for such quantitative integration have remained unclear. We adopted a simple strategy by assuming that changes in mRNA could be directly converted into changes in model parameters, as previous studies have done to examine phenomena such as treatment of colorectal cancer (Hector et al., 2012), electrophysiology of failing ventricular myocytes (Walmsley et al., 2013), and sex differences in arrhythmia susceptibility (Yang et al., 2017). Our work builds on these prior studies and takes a step forward by simulating, and then validating, predictions of how individual cell lines respond to secondary insults.

### The use of iPSC-CMs as an experimental model for cardiotoxicity caused by cancer therapeutics

In addition to mechanistic mathematical modeling, a second important aspect of our study was the use of iPSC-CMs as a model system for the study of cardiotoxicity. Despite the fact that iPSC-CMs exhibit an “immature” phenotype compared with adult cardiac myocytes, the human origin of these cells provides advantages compared with animal experiments, as iPSC-CMs contain the same processes and pathways as adult myocytes (Gintant et al., 2019). Several recent studies have demonstrated the utility of iPSC-CMs for elucidating mechanisms of cardiotoxicity caused by cancer therapeutics, including anthracyclines (Holmgren et al., 2015; Burridge et al., 2016) and more recently TKIs (Jacob et al., 2016; Sharma et al., 2017; Wang et al., 2019). These studies have shown the value of the iPSC-CM model for allowing both Omics assays, such as gene expression and protein expression, and physiological assays, such as action potentials (Sharma et al., 2017), contractile force (Jacob et al., 2016), or oxygen consumption (Wang et al., 2019). These investigations have provided important novel insight into potential cardiotoxic mechanisms, such as the suggestion that insulin-induced signaling may be protective when specific pathways are inhibited by TKIs (Sharma et al., 2017), and the finding that downregulation of oxidative phosphorylation may contribute to sorafenib-induced cardiotoxicity (Wang et al., 2019). Our study improves upon this prior work by rigorously linking the Omics to the physiology through simulations with mechanistic models. This strategy allowed us to: 1) examine changes in cellular behavior that resulted from drug-induced changes in gene expression rather than from direct block of ion channels; 2) effectively prioritize the low-throughput physiology experiments; and 3) examine mechanisms underlying divergent predictions between the two cell lines. An important improvement that needs to be made in this area, however, is the more widespread adoption of engineered cell culture systems exhibiting a reproducible and mature phenotype, as drug responses in these systems are more likely to mimic those seen in adult patients. Efforts to improve maturity of the cellular system (Charwat et al., 2022; Huebsch et al., 2022) can be combined with computational approaches to translate drug responses across cells with different properties (Gong and Sobie, 2018; Tveito et al., 2018; Jaeger et al., 2019), thereby providing additional confidence in the results.

### Quality control issues in iPSC-CM experiments and steps taken to promote reproducibility

iPSC-CMs have generated considerable enthusiasm in recent years as a valuable model system for examining drug-induced pathophysiology (Sharma et al., 2017; Blinova et al., 2018; Gong and Sobie, 2018). Experiments using iPSC-CMs, for instance, are an integral part of the Comprehensive *in vitro* Proarrhythmia Assay (CiPA) initiative, which aims to improve the testing of drugs for potential risk of Torsades de Pointes due to ion channel block (Fermini et al., 2016; Blinova et al., 2018). When iPSC-CMs are used for drug screening purposes, however, it is important to have appropriate quality control steps implemented to ensure the reliability of the differentiation and the quality of the cells. For this study, we differentiated iPSC-CMs in house, using well-established procedures (Kattman et al., 2011) and a metabolic selection step (Tohyama et al., 2013) to generate preparations with approximately 95% cardiomyocytes. To validate purity, we assessed each differentiation by flow cytometry for the cardiac-specific membrane protein SIRPA (Dubois et al., 2011). During physiological experiments, we implemented additional quality control steps to verify that myocytes would exhibit similar behavior across the different batches of cells generated over the course of the study. Specifically, we performed electrophysiology recordings only when cells met following criteria: 1) no apparent blebbing of the cells anywhere on the coverslip; 2) cells were plated in close proximity with total cell count of approximately 35,000–40,000; and 3) cells beat spontaneously at 37°C. These quality control steps ensured that we obtained reproducible results, even though multiple iPSC-CM differentiations were performed from the same original donor iPSC clones, sometimes several months apart. Arrhythmia susceptibility to hypokalemia, for instance, was assessed with both CaT and AP recordings, in different experimental series, using cells from multiple differentiations. The remarkable concordance of these two sets of experiments (Supplementary Figure S11) provides reassurance that the results we obtained were not specific to individual iPSC-CM differentiations.

## Limitations and future work

Besides the issue of iPSC-CM maturity mentioned above, several of the study’s limitations suggest avenues that can be pursued in future research on TKI-induced cardiotoxicity. For instance, the expense of comprehensive mRNA sequencing forced us to test only a single concentration of each TKI, with that concentration determined based on estimated plasma concentrations in patients following standard dosing (van Hasselt et al., 2020). Although the lack of concentration-response data is a limitation at present, the experimental and computational pipeline we outlined demonstrates how individual drugs can be prioritized for in depth follow-up studies. Another limitation is the fact that the model outputs are limited to changes in electrophysiology, Ca^2+^ handling, and contraction. Although this model allowed for straightforward tests of experimental predictions, it only simulates a small part of myocyte biology. As more information becomes available, additional reactions can be quantitatively considered, which will allow for the development of an integrated, multiscale mathematical model that also simulates other processes potentially involved in drug-induced cardiotoxicity, such as hypertrophy (Ryall et al., 2012) and apoptosis (Shin et al., 2014). Examination of such a larger model that can simulate cross talk between pathways would provide the opportunity to identify targets that can potentially mitigate cardiotoxicity, which may allow for the design of combination therapies in which a second drug ameliorates the cardiotoxic effects of a TKI.

Finally, we should note that although the differences that were predicted and observed between the two cell lines are intriguing, a much more comprehensive study will be required to characterize drug responses across a population. The strategy we have pursued should help provide a road map for how broad coverage can be balanced with mechanistic rigor in such an investigation.

## Conclusion

In summary, we have integrated transcriptomic data with mathematical modeling and physiological assays to elucidate individual-specific responses to TKI-induced EC coupling abnormalities. This study is a step forward for individualized drug prescription that can potentially minimize the probability of drug-induced cardiotoxicity.

## Data Availability

The datasets presented in this study can be found in online repositories. The names of the repository/repositories and accession number(s) can be found below: https://www.ncbi.nlm.nih.gov/geo/, GSE217421.
