# Supplementary material for: Predicting individual-specific cardiotoxicity responses induced by tyrosine kinase inhibitors
Source: Front Pharmacol. 2023 Apr 10;14:1158222. doi: 10.3389/fphar.2023.1158222 (PMC10123273; doi:10.3389/fphar.2023.1158222)
Supplement: Supplementary file 1 [file DataSheet1.PDF]

SUPPLEMENTAL MATERIALS

Transcriptomics based mechanistic modeling elucidates individual-specific cardiotoxicity induced by tyrosine kinase inhibitors

Jaehee V. Shim, Priyanka Dhanan, Rafael Dariolli, Evren Azeloglu, Yuguang Xiong, Bin Hu, Gomathi Jayaraman, Christoph Schaniel, Ravi Iyengar, Nicole Dubois, Eric A. Sobie

Correspondence: eric.sobie@mssm.edu

SUPPLEMENTAL METHODS

Reprogramming of skin fibroblasts to generate iPSCs

The study has been reviewed and approved by the Icahn School of Medicine at Mount Sinai Institutional Review Board (IRB). The initial step in the study was to generate iPSCs from two healthy volunteers. Subject A was a 36 year-old, white, non-Hispanic female and subject B was a 25 year-old, white, non-Hispanic female. Prior to participation, cardiovascular health of the subjects was confirmed via full electrocardiogram and blood pressure measurements. iPSCs were reprogrammed from dermal fibroblasts. Fibroblasts were isolated from 3 mm skin punch biopsy samples of the inner arm that were cultured in DMEM supplemented with 20% FBS at 37ºC with 5% CO_2 for one week before being passaged. Cells were reprogrammed before their third passage using mRNA with microRNA boost method as previously described.[1] A detailed experimental protocol is available on the institutional website (see list of Standard Operating Procedures below).

Differentiation of iPSCs to cardiomyocytes

On day 0 of differentiation, embryoid bodies (EBs) were generated from iPSC lines using collagenase B (Roche). EBs were collected after 1h and resuspended in RPMI 1640 base media (ThermoFisher) containing 0.5X B27 supplement (ThermoFisher), 2 mM glutamine (Gibco-BRL), 1 mM ascorbic acid (Sigma), and 4 x 10^{-4} M monothioglycerol (Sigma) with BMP4 (2 ng/ml, R&Dsystem) and Thiazovivin (2 µM, Millipore). On day 1, EBs were harvested and resuspended in induction medium (base media with basic fibroblast growth factor (bFGF; 2.5 ng/ml, R&Dsystem), Activin A (20 ng/ml, R&Dsystem) and BMP4 (20 ng/ml, R&Dsystem)). On day 3, the EBs were harvested and resuspended in base media with vascular endothelial growth factor (VEGF; 5 ng/ml, R&Dsystem) and XAV939 (10 µM, Stemolecule). On day 5, EBs were harvested and resuspended in base media with vascular endothelial growth factor (VEGF; 5 ng/ml, R&Dsystem). On day 10 and thereafter, EBs were cultured in base media with media change every 4 days until day 30 for further analysis. On day 20 EBs were harvested and incubated in collagenase type II (1 mg/ml; Worthington) in Hanks solution (in mM: NaCl 136; NaHCO3 4.16; NaPO4 0.34; KCl 5.36; KH2PO4 0.44; dextrose, 5.55 mM; HEPES, 5 mM) overnight at 37 °C. After incubation EBs were pipetted gently to dissociate the cells, washed, and resuspended in base media. Cells were plated at 1 x 10^6 cell per well of a 6-well plate and

subjected to lactate selection as previously described.[2] Selected cells were incubated in collagenase type II (1 mg/ml; Worthington) in Hanks solution (in mM: NaCl 136; NaHCO3 4.16; NaPO4 0.34; KCl 5.36; KH2PO4 0.44; dextrose, 5.55 mM; HEPES, 5 mM) overnight at 37 °C. After incubations, cells were harvested, spun, and resuspended in base media with Thiazovivin (2 µM, Millipore). 35,000 cells were plated onto Matrigel-covered glass cover slips for calcium transient and action potential analysis. 250,000 cells were plated per one well of a 24-well plate for drug treatment. Media was changed to base media without Thiazovivin (2 µM, Millipore) the day after plating.

Flow Cytometry analysis

EBs were incubated in collagenase type II (1 mg/ml; Worthington) in Hanks solution (NaCl, 136 mM; $NaHCO_3$, 4.16 mM; $NaPO_4$, 0.34 mM; KCl, 5.36 mM; KH_2PO_4, 0.44 mM; dextrose, 5.55 mM; HEPES, 5 mM) overnight at 37 °C. The EBs were pipetted gently to dissociate the cells, washed, resuspended in staining solution (PBS with 0.1% BSA) and filtered. Antibodies were diluted in staining solution, and cells were resuspended in antibody solution and stained on ice for 30 min. Cells were washed and resuspended in staining solution for analysis. Cell counts were collected using a LSRII (BD Biosciences) and data were analyzed using FlowJo software. The following antibodies and dilutions were used: anti-human CD172a/b (SIRPα/β) (Biolegend; clone SE5A5; 1:200) and anti-human CD90 (Biolegend, clone 5E10; 1:200).

Immunofluorescence analysis

EBs were incubated in collagenase type II (1 mg/ml; Worthington) in Hanks solution (NaCl, 136 Mm; $NaHCO_3$, 4.16 mM; $NaPO_4$, 0.34 mM; KCl, 5.36 mM; KH_2PO_4, 0.44 mM; dextrose, 5.55 mM; HEPES, 5 mM) overnight at 37 °C. The EBs were pipetted gently to dissociate the cells, washed, resuspended in staining solution (PBS with 0.1% BSA), then filtered. Cells were plated on Matrigel-covered glass cover slips in base media, and cultured for 5 days. Slides were fixed for 10 min in 4% PFA and incubated for 1 h in blocking solution (PBS with 0.1%Triton and 1% BSA). Primary antibodies were diluted in blocking solution added to slides for 1 h at room temperature or overnight at 4°C, followed by 3 washes in blocking solution. Secondary antibodies were diluted in blocking solution and added to slides for 1 h at room temperature. Slides were washed 3 times with blocking solution, counterstained with DAPI and mounted

using glycerol-based nPG (Sigma-Aldrich, P3130) antifade mounting media. The following primary antibodies and dilutions were used: anti-cTnT (ThermoSci, 1:300); anti-Mlc2v (Proteintech, 1:100), anti-CX43 (Sigma-Aldrich, 1:100) and anti-alpha-actinin (Sigma-Aldrich, 1:400). Secondary antibodies conjugated with Alexa dyes were obtained from Jackson Immunoresearch and used at 1:500. Fluorescence images were obtained using either Leica DM6000 or DM5500B microscopes. Images were processed using FIJI ImageJ, or Adobe Photoshop software.

iPSC-CM drug treatment and mRNA harvesting

iPSC-CMs from each subject were treated with 26 FDA approved TKIs at an estimated maximal clinical concentration for 48 hours. Information on dilution of each TKI is provided in Supplementary Table S1. After drug treatment, total RNA was collected for RNA-seq using TRIzol (15596018, Life Technologies). Each drug-treated condition had four biological replicates, and vehicle-treated controls had 6-8 replicates.

Quantification of drug-induced changes in gene expression

Isolated mRNA quality was assessed using a Fragment Analyzer with a high sensitivity RNA analysis kit (Advanced Analytical Technologies, Cat: DNF-472). Only samples with an RNA integrity number of 7.0 (out of 10) proceeded to library preparation and sequencing.

The reference sequence utilized was UCSC hg38 (downloaded in Dec. 2017) from UCSC genome sequence database (https://hgdownload.soe.ucsc.edu/downloads.html#human). Annotation for hg38 can be found at: http://hgdownload.cse.ucsc.edu/goldenPath/hg38/database/ncbiRefSeqCurated.txt.gz.

Additional experimental details in Standard Operating Procedures documents

All experiments in this study were performed by investigators within the Drug Toxicity Signature Generation Center at Icahn School of Medicine at Mount Sinai. Additional details for particular experimental methods are provided in Standard Operating Procedures (SOPs) published by the center, www.dotxs.org. Relevant SOPs include:

DToxS SOP CE – 4.0: Drug Treatment and Cell Lysis

DToxS SOP CE – 6.0: Skin Biopsy Punch Explant Culture for Derivation of Primary Human Fibroblasts

DToxS SOP CE – 7.0: Reprogramming Human Fibroblasts to Human Induced Pluripotent Stem Cells (hiPSCs) using the mRNA with microRNA boost method

DToxS SOP A – 1.0: Total RNA Isolation

DToxS SOP A – 2.0: Protein Isolation

DToxS SOP A – 6.0: High-throughput mRNA Seq Library Construction for 3' Digital Gene Expression (DGE)

DToxS SOP A – 7.0: Sequencing 3'-end Broad-Prepared mRNA Libraries

DToxS SOP CO – 3.1: Generation of Transcript Read Counts

Mechanistic mathematical model modifications

The mathematical model employed to generate predictions in the current study was built by combining a mathematical model of iPSC-CM electrophysiology and intracellular [Ca^{2+}][3] with a model of myofilament interactions that was originally developed for adult rat ventricular myocardium.[4] A schematic illustrating the processes included in this model is shown in Supplementary Figure S1.

To compensate for systolic [Ca^{2+}] levels that are lower in iPSC-CMs than in adult ventricular myocytes, we adjusted the myofilament model to make the contractile machinery more sensitive to Ca^{2+}. To do this, we multiplied the default value of k_{on} by 4000 and *troponin C* concentration by 1000, both of which are important for the dynamics of calcium binding to troponin for thin filament regulation. .

Integration of actin and myosin gene expression to predict iPSC-CM contraction

For MHC, we used both β and α isoforms, encoded by *MYH7* and *MYH6* respectively. The ratio of α-MHC to β-MHC is indicative of iPSC-CMs maturity in which lower measure indicate higher maturity {Reiser, 2001 #1693}. As described later in the Supplemental Materials, our gene expression data indicated that our cells had abundant β-MHC expression and low α-MHC expression, exhibiting relatively high maturity for iPSC-CMs.

Incorporation of actin and myosin fold changes into the mathematical model was performed by introducing additional parameters in the equation describing cross-bridge formation dynamics. We assumed that up- or downregulation of both actin and myosin would promote or inhibit cross bridge formation, respectively, but opposing effects on actin and myosin could potentially cancel each other out. To implement this idea, we computed the product of actin and myosin fold change values. This scale factor, describing combined effects on both actin and myosin, was the used to modify the model parameter $perm_{50}$, which acts as an EC50 value for the nonlinear equation describing nearest neighborhood cooperativity of regulatory units for cross-bridge formation.

$$Scalefactor_{actinMyosin} = 1 + \omega\left(\frac{[actin \times myosin]_{TKI}}{[actin \times myosin]_{CTRL}} - 1\right), \omega = 0.5$$

$$Perm50_{new} = \frac{Perm50_{default}}{Scalefactor_{actinMyosin}} \;, Perm50_{default} = 0.5$$

$$permtot = (1/(1 + \frac{perm50_{new}}{Trop\_regulatory(x)}^{nperm}))0.5$$

Supplementary Figure S2 displays the validations we considered to ensure that the assumptions underlying this approach were reasonable. First, we examined the distribution of the actin and myosin product values to make sure it is not skewed (Supplementary Figure S2). Next, we computed fold changes of the product of actin and myosin. We explored different version of integrating this value by adding a scaling step with a constant labeled ω as shown below (Supplementary Figure S2). The additional scaling was necessary to minimize the skewedness of the SL shortening output distribution (Supplementary Figure S2). Finally, we verified that if the cooperativity change based on actin and myosin product value is in line with our original assumption by examining both *permtot* and SL shortening output (Supplementary Figure S2).

Calculation of Arrhythmic Index

To predict how iPSC-CMs treated with TKIs would respond to potentially arrhythmogenic secondary insults, we simulated 3 perturbations: (1) an increase in L-type Ca^{2+} current I_{CaL}; (2)

block of rapid delayed rectifier current I_{Kr}; and (3) hypokalemia. To synthesize results obtained with all 3 insults, we calculated an "Arrhythmic Index" (AI) based on the Euclidean "distance" from the unperturbed state, in a 3-dimensional space. In this case, origin represents smaller magnitude of insult which means that if a TKI is closer to origin, it translates into greater proarrhythmic potency of the drug. To make the comparison simpler and more intuitive, we normalized the Euclidean distance of each TKI by the control distance and negative log transformed to center the indices around zero and to arrive at the metric where large positive number represents higher risk.

$$EuclideanDistance_{Condition} = \sqrt{(X^2 + Y^2 + Z^2)},$$
$$where\ X, Y, Z\ corresponds\ to\ threshold\ values$$

$$AI = -log(\frac{EuclideanDistance_{Drug}}{EuclideanDistance_{CTRL}})$$

Cell plating, dye loading, and solutions for physiological recordings

Five days after lactate selection to increase purity of iPSC-CM cultures, cells were plated on Matrigel-coated coverslips (83.1840, SARSTEDT) and treated with either DMSO (vehicle) or TKI, at the same concentration used for gene expression measurements. After 48 hour drug treatment, cells were loaded with either fluo-3-AM for Ca^{2+} transient recordings (10 μM, 30 minutes, Biotium #50013) or FluoVolt for action potential recordings (Life Tech F10488, incubation for 30 min according to manufacturer's protocol, no concentration provided). Dyes were added to modified Tyrode's solution containing 140 mM NaCl, 5.4 mM KCl, 10 mM HEPES, 1 mM NaH_2PO_4, 1 mM $MgCl_2$, 2 mM $CaCl_2$, and 5 mM glucose (pH 7.4).

Baseline physiology recordings were obtained while cells were paced at 1 Hz and perfused with modified Tyrode's solution at 37°C. Measurements were made with a Zeiss Exciter confocal microscope operating in line-scan mode, with argon laser excitation at 488 nm and emission recorded through a 505 nm longpass filter. Tyrode's solutions used for action potential recordings contained 10 μM blebbistatin (B0560, Sigma) to immobilize membrane movement.

Membrane movement assessment

For assessment of contraction, recordings made on iPSC-CMs loaded with FluoVolt, in the absence of blebbistatin. In line scan recordings during 1 Hz pacing at 5.4 mM extracellular [K^+], a bright spot of iPSC-CM membrane was tracked to monitor membrane movement.

CaT, AP, and SL metric computation

All data collected were processed and analyzed using custom MATLAB scripts. The following metrics, illustrated in Supplementary Figure S3, were computed to describe CaT and AP morphology: decay tau, CaT AUC, AP and CaT triangulation and membrane movement. Decay tau was computed by measuring calcium decay time from half maximal CaT to resting [Ca^{2+}] as highlighted in figure 4A. The total calcium transient, or CaT AUC, was computed using MATLAB function, *trapz,* which performs numerical integration. CaT triangulation was calculated as $Duration_{90}/Duration_{50}$, whereas AP triangulation was calculated as $Duration_{90} - Duration_{50}$. Membrane movement was calculated as the displacement of dye-loaded membrane between resting and the fully contracted state (Fig. 2B).

Figures related to Supplemental Methods:

**Fig. #**	**Description**
S1	Mathematical model schematic
S2	Explanation of how to convert from actin & myosin gene expression measurements to mathematical model parameters
S3	Metrics extracted from time course simulations and measured experimentally
S4	Integration of individual ionic currents to compute differences between drug-treated and untreated myocytes

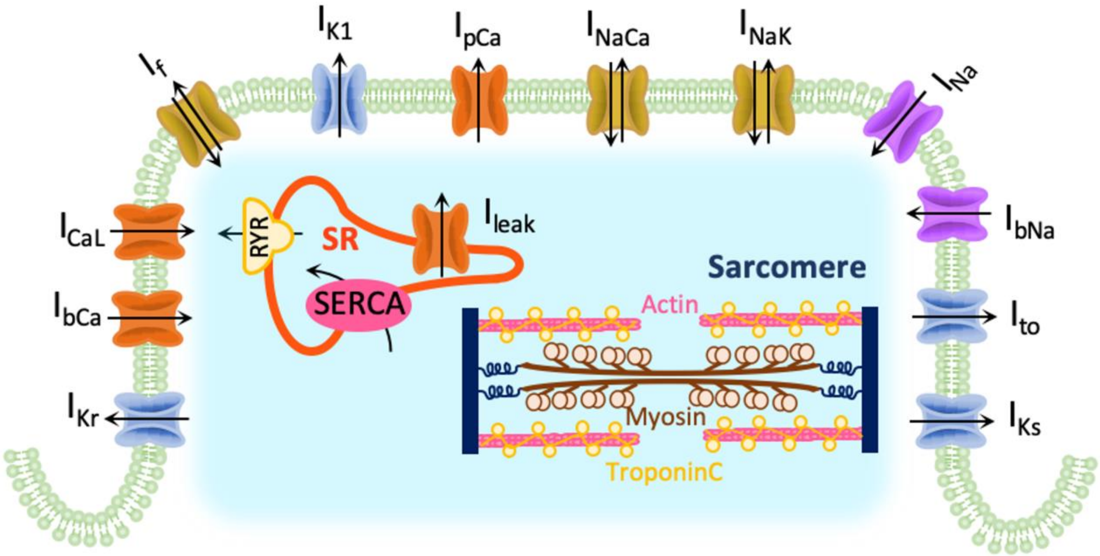

Fig. S1. Integrated model used in simulations. The mathematical model combined the representation of iPSC-CM electrophysiology and intracellular [Ca^{2+}] from Paci et al{Paci, 2013 #1568} and the myofilament model of Rice et al.{Rice, 2008 #1644}.

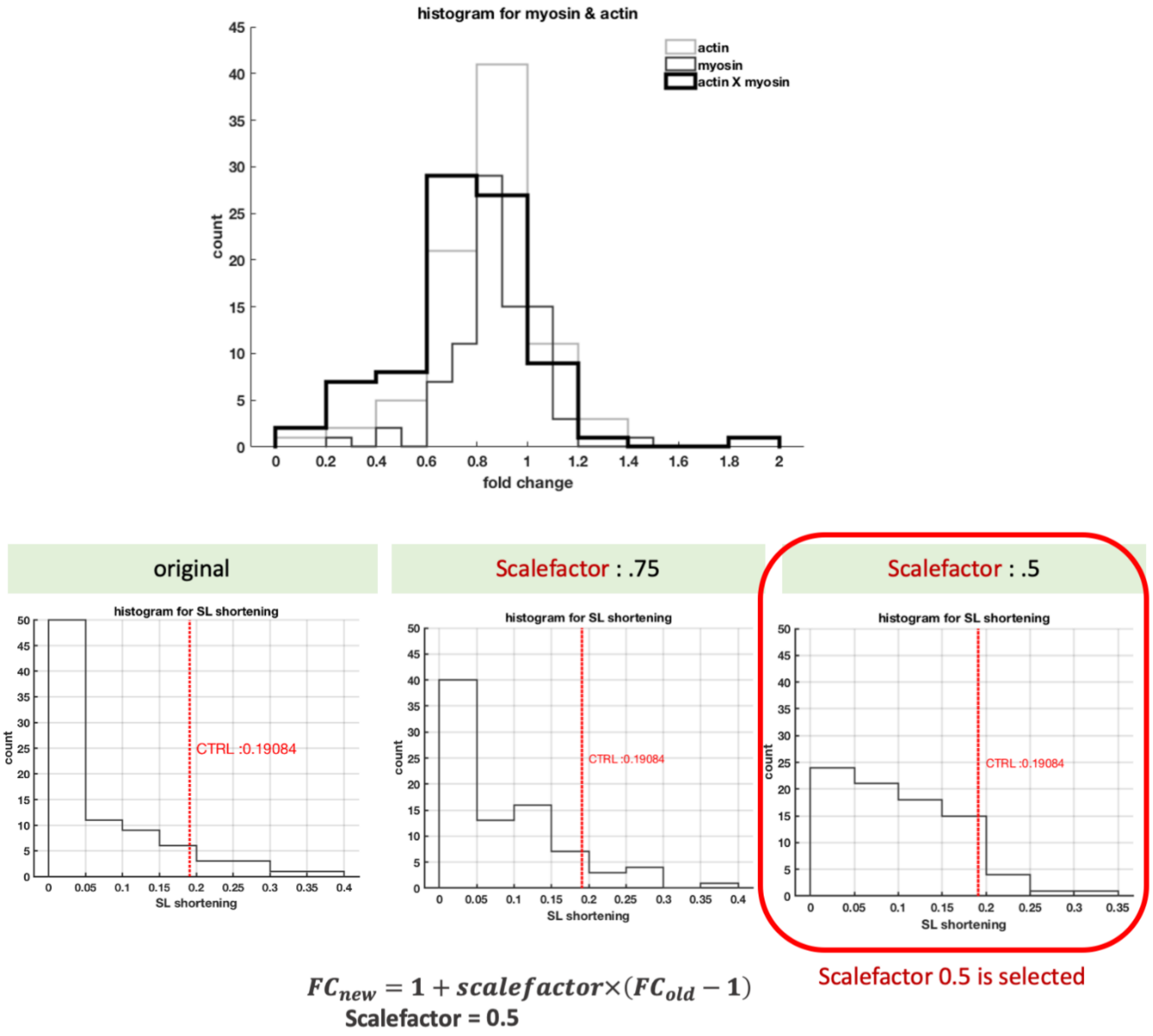

Fig. S2. Conversion from gene expression measurements to a model parameter scaling factor for actin-myosin interactions Gene expression for actin and myosin are used to compute a scaling factor for cross-bridge cooperativity. **(A)** First, fold change values for actin and myosin were multiplied to generate single metric to use for scaling factor. The distribution of actin, myosin, and the product of the two are shown. **(B)** Using the product of actin and myosin, SL shortening distance were computed for all conditions to examine the distribution. The product value generated extremely skewed SL shortening population, showing that scaling cooperativity with the original product value was not able to differentiate most of the simulation conditions. To improve the qualitative comparison the 50 counts of events that were under 0.05 µm of SL shortening, we scaled the product value further using 75% and 50% of the original number. Of the two conditions, using 50% of the original product improved the distribution significantly. **(C)** Distribution of myosin and actin fold change product (FC original), the 75% and 50% of the FC original, and 50% are shown in relation to the SL shortening simulations each generated.

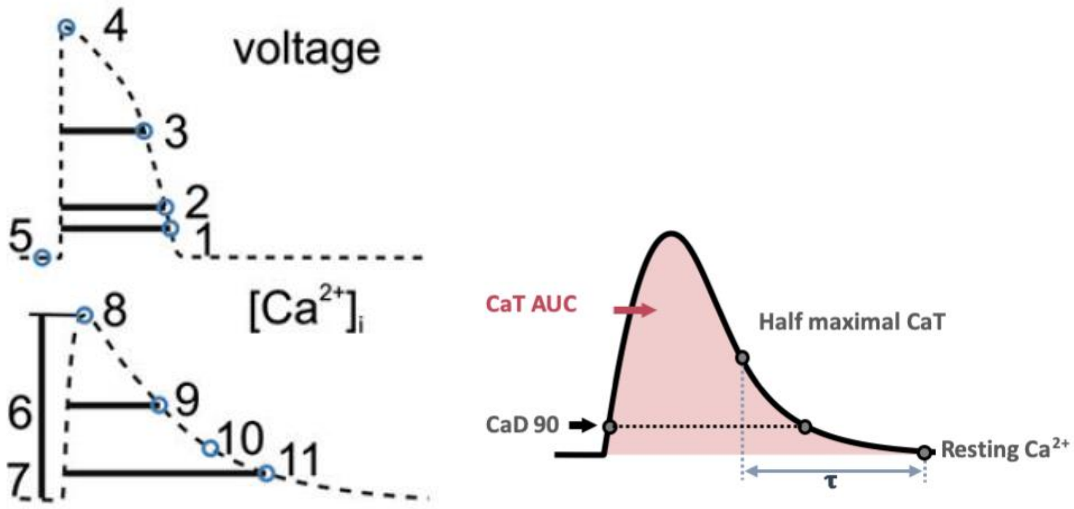

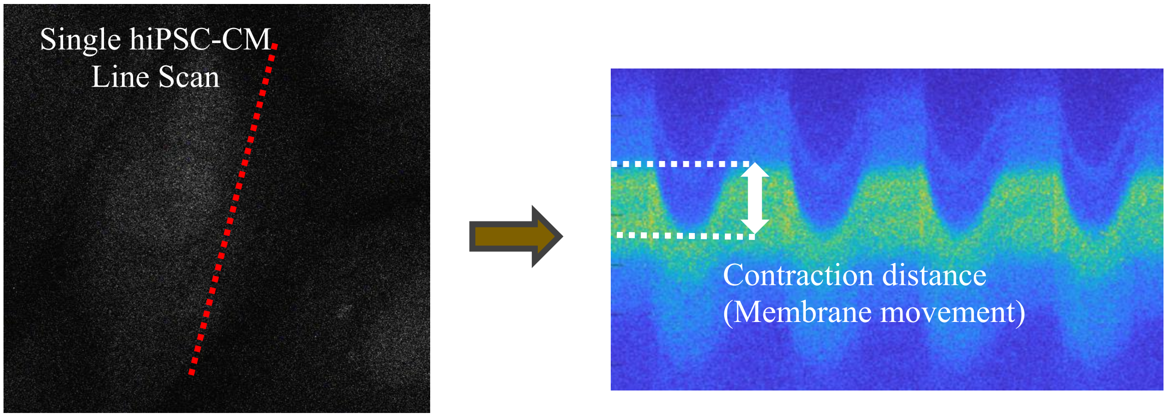

Fig. S3. Metrics calculated from simulation and experimental results. From fluorescent recordings of action potentials and intracellular [Ca^{2+}], we computed metrics as illustrated, including duration at 50% and 90% of recovery ($APD_{50/90}$ and $CaTD_{50/90}$, respectively) decay time constant for [Ca^{2+}] recordings (Decay Tau), and area under the curve (AUC) for [Ca^{2+}] recordings. Identical metrics were calculated from both simulation time courses and experimental recordings, allowing for direct comparison. For sarcomere length (SL) shortening, we generated SL shortening predictions as a metric for TKI induced contractility dysfunction. To test these experimental predictions, we recorded an indirect measure that we call "membrane movement". Importantly, these numbers, mostly derived from waveform durations, did not require precise calibration of experimental recordings.

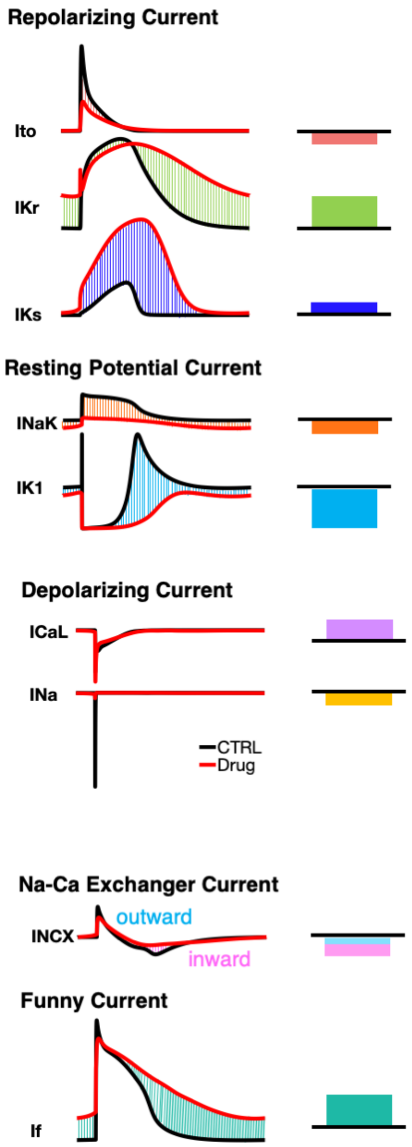

Fig. S4. Integration of individual ionic currents to calculate differences is total charge carried. To examine the mechanisms through which alterations in ionic current expression contributed to arrhythmia risk, we calculated simulated current waveforms in untreated (black) and TKI-treated iPSC-CMs. For each current we computed the difference in total charge (ΔQ) carried by each current from the beginning to the end of the action potential, as shown. A positive ΔQ encourages membrane repolarization and is considered anti-arrhythmic whereas negative ΔQ can lead to long action potentials and is considered pro-arrhythmic.

SUPPLEMENTARY RESULTS

Supplemental Results are summarized in the chart below. Most supplementary figures display additional results to complement the limited results discussed in the main manuscript text. For each figure, therefore, the corresponding subsection of the main text Results is indicated.

Fig. #	Description	Results subsection	Significance
S5	Ranking of how all TKIs influence AP triangulation	Simulations predict individual-specific alterations to AP, CaT, and SL shortening waveforms	Simulations produce comprehensive prediction of how all TKIs affect APs in both cell lines.
S6	Ranking of how all TKIs influence contraction	Simulations predict individual-specific alterations to AP, CaT, and SL shortening waveforms	Simulations produce comprehensive prediction of how all TKIs affect contraction in both cell lines.
S7	Comparison of simulation predictions and experimental tests	Cellular physiology experiments confirm individual-specific drug responses	Simulation predictions and experimental results are compared in Fig. 3. This figure shows additional data not presented in Fig. 3.
S8	Predictions of increased or decreased susceptibility to secondary insults	Secondary insults can potentiate TKI-induced arrhythmogenicity	Simulations produce comprehensive prediction of how all TKIs influence cellular susceptibility to hypokalemia in both cell lines.
S9	Hypokalemia predictions and experimental tests: Ca^{2+} transient measurements	Action potential and intracellular [Ca^{2+}] recordings validate the two-hit hypothesis with hypokalemia as the secondary insult	Experimental metrics derived from [Ca^{2+}] waveforms, in cells treated with various TKIs, as a function of extracellular [K^+], in both cell lines.
S10	Hypokalemia predictions and experimental tests: action potential measurements	Action potential and intracellular [Ca2+] recordings validate the two-hit hypothesis with hypokalemia as the secondary insult	Experimental metrics derived from [Ca^{2+}] waveforms, in cells treated with various TKIs, as a function of extracellular [K^+], in both cell lines.
S11	Arrhythmia percentage calculated from Ca^{2+} or action potential experiments	Action potential and intracellular [Ca^{2+}] recordings validate the two-hit hypothesis with hypokalemia as the secondary insult	Arrhythmia susceptibility was determined in separate experiments that measured either membrane voltage or intracellular [Ca^{2+}], with similar results obtained
S12	Cell line-dependent effects on ionic currents	Simulations reveal that drugs may increase arrhythmia susceptibility through downregulation of repolarizing ionic currents	Examination of changes in ionic currents caused by TKIs can provide mechanistic explanations for iPSC-CM susceptibility.
S13	The curious case of trastuzumab	Simulations reveal that drugs may increase arrhythmia susceptibility through downregulation of repolarizing ionic currents	In Cell Line B, trastuzumab causes unusual, non-monotonic effects as a function of extracellular [K^+], and simulations provide potential mechanistic explanations.

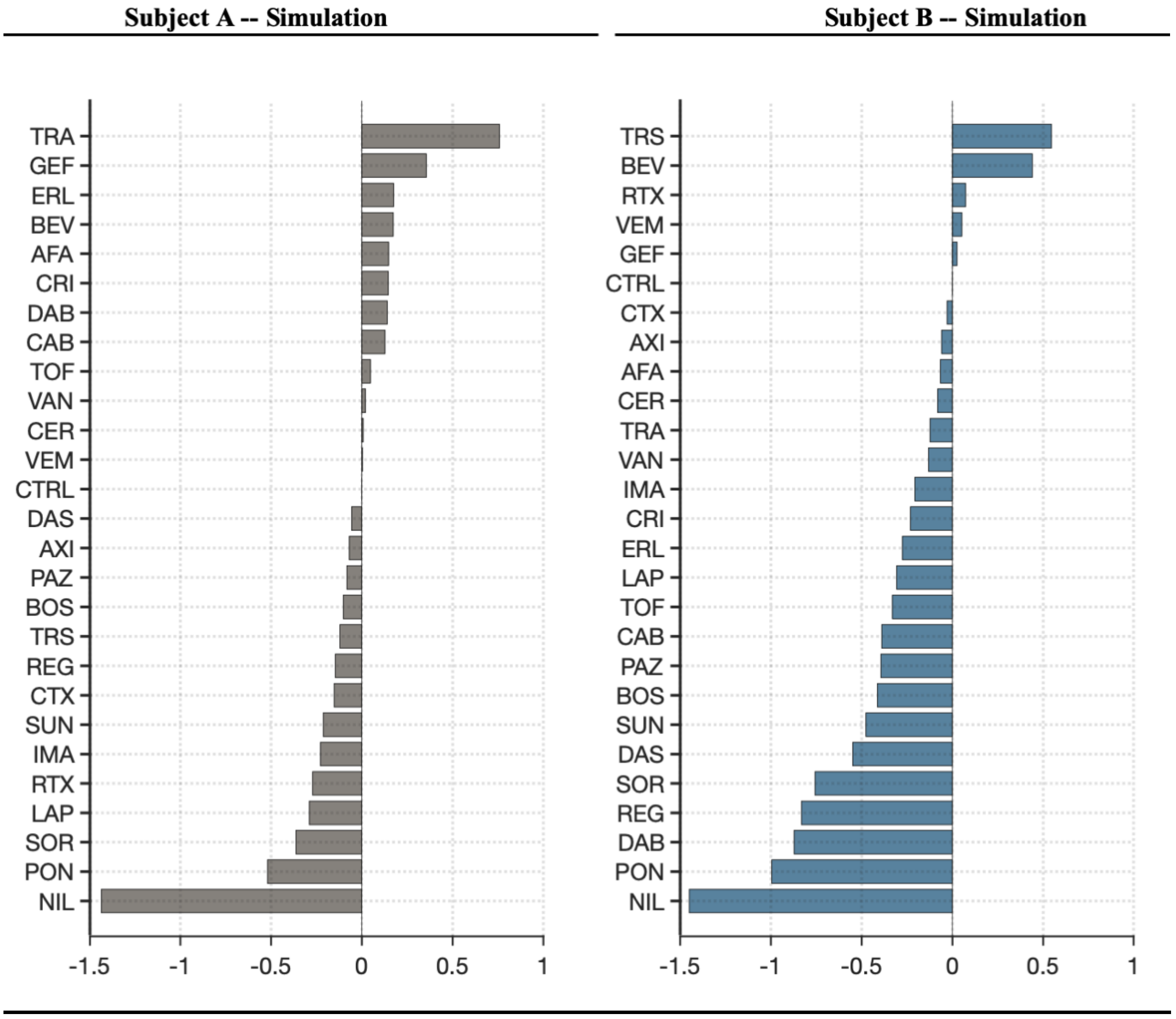

Fig. S5. Predictions of AP triangulation across all drugs tested. AP triangulation metric is computed for all 26 TKIs tested and ordered so that the most proarrhythmic TKIs are at the top.

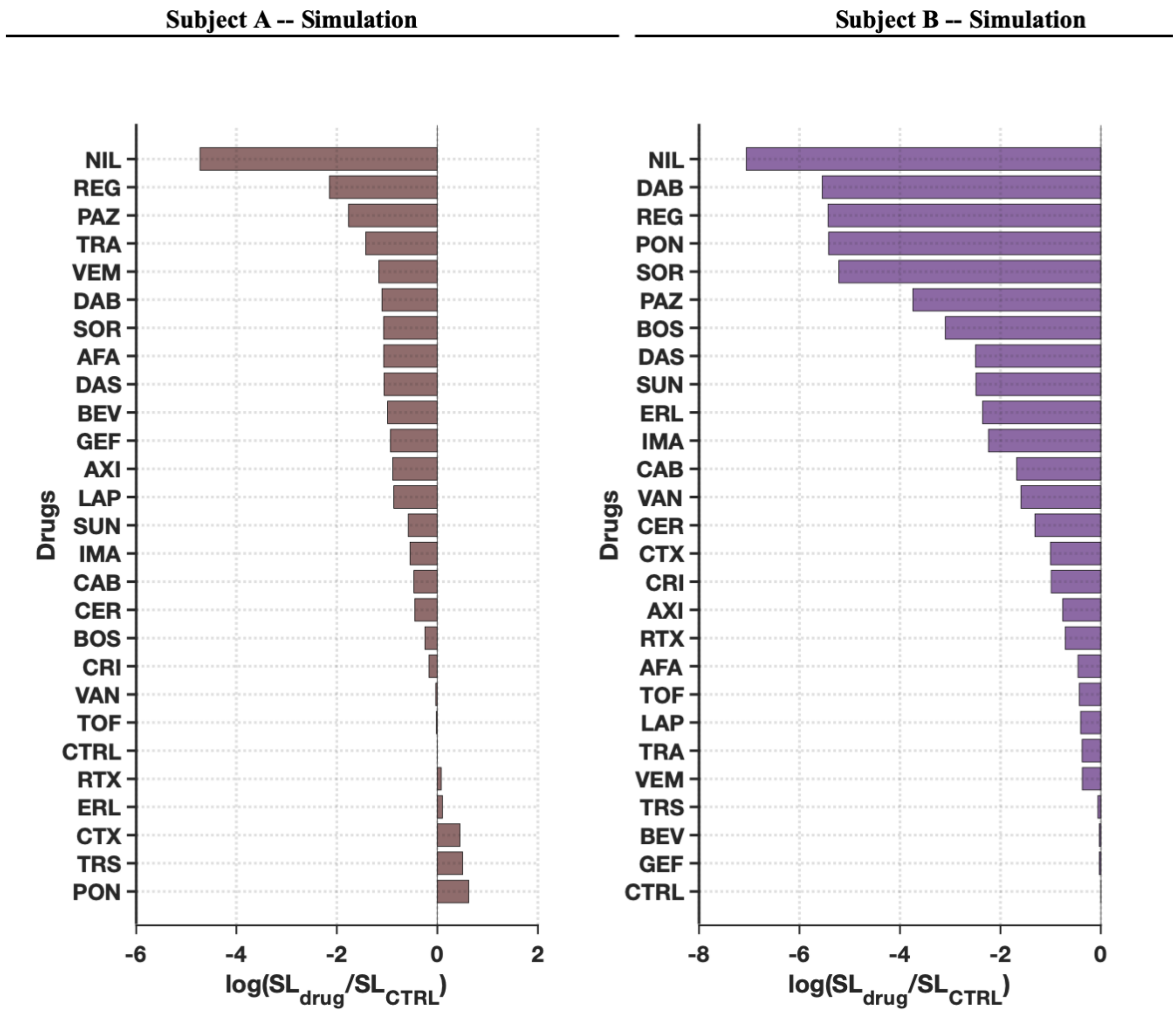

Fig. S6. Predictions of changes in iPSC-CM contraction across all drugs tested. SL shortening distance is computed for all 26 TKIs tested and ordered such that cells with the largest decrease in contraction strength are at the top.

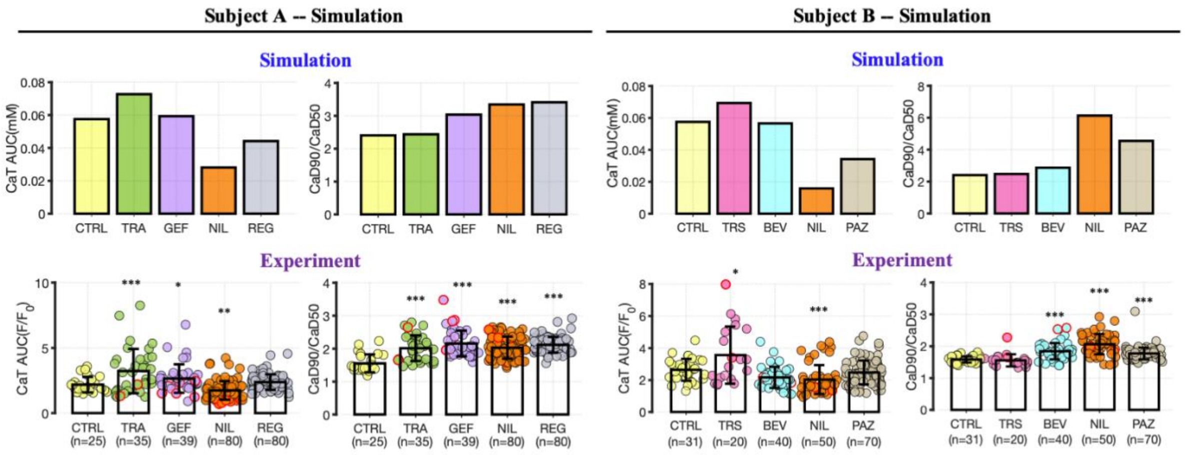

Fig. S7. Comparison of simulation predictions and experimental tests. In the main text, Figure 3 compares simulation and experimental results for Decay Tau and SL shortening. This figure shows results for CaT area under the curve (AUC) and CaT triangulation, with a similar format. Figure 3D in the main text compares experimental and simulation results for all experiment-simulation comparisons, including those shown here. Error bars indicate standard deviation, and asterisks indicate significantly different from control, based on two sample, unpaired *t* test (*$P < 0.05$, **$P < 0.01$ and ***$P < 0.001$).

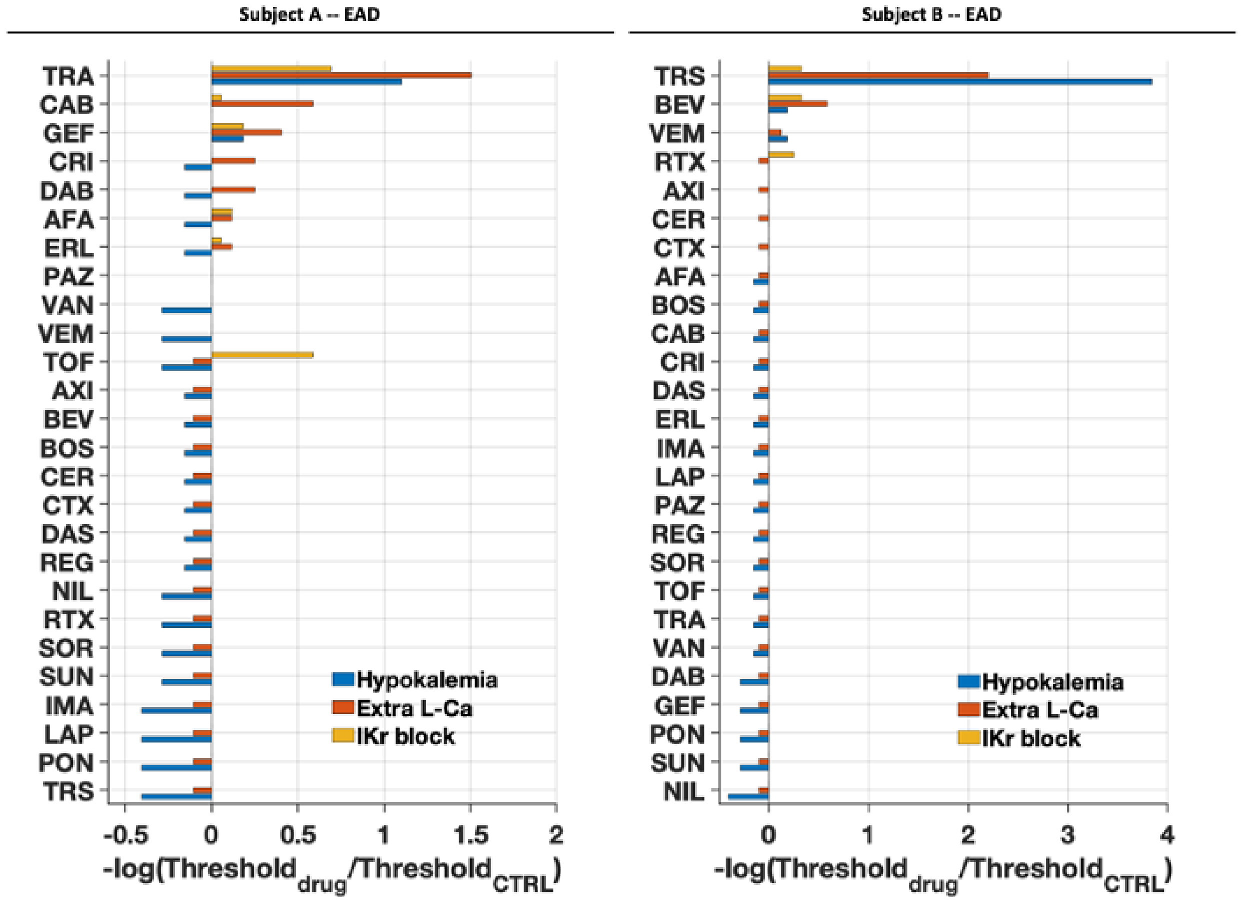

Fig. S8. Predictions of increased or decreased arrhythmia susceptibility due to all arrhythmic perturbations. Each of the 3 arrhythmic perturbations was applied under progressively increasing degree of secondary insult until the AP traces show arrhythmic behavior. Threshold is defined as the severity of the secondary insult at which the TKI-induced proarrhythmic potency is realized. For each drug and each insult, a predicted change in arrhythmia susceptibility can be computed by comparison with the untreated iPSC-CMs. Figure 4 in the main text shows the most highly ranked (increased susceptibility) and lowest ranked (decreased susceptibility) TKIs in the two cell lines, with hypokalemia as the secondary insult. This figure shows the results across all TKIs and all secondary insults, in both cell lines. Arrhythmogenic Index is computed as a weighted average across the three secondary insults.

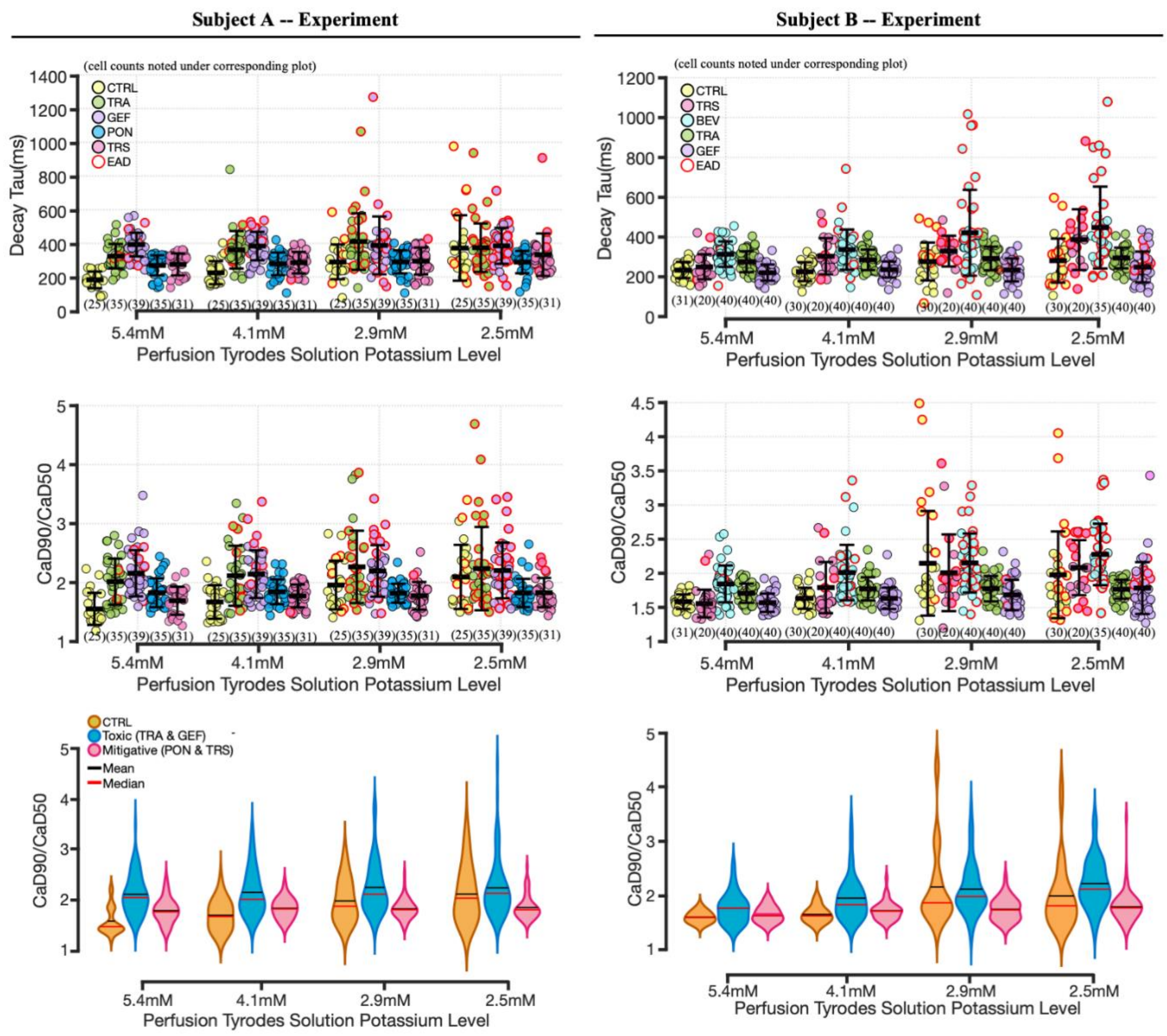

Fig. S9. Hypokalemia predictions and experimental tests: Ca^{2+} transient measurements. Experimental results are shown as a function of extracellular $[K^+]$, for 4 drugs, 2 cell lines, and two proarrhythmic markers: Decay Tau and CaT triangulation (CaD_{90}/CaD_{50}). All recorded data points are shown, with superimposed black lines to indicate means ± **σ**. Two way, unbalanced ANOVA results show, for both subjects, TKI treatment and hypokalemia increase the value of decay tau and CaD_{90}/CaD_{50}. For subject A, ANOVA reported significant interaction between TKI treatment and hypokalemia for both metrics whereas for subject B, it was only reported for tau decay (cut off $P < 0.05$). In the two bottom panels, the experimental data are grouped as 'toxic' and 'mitigative' based on the prediction result (Figure5). For both subjects, toxic group shows significant increase in both decay tau and CaT_{90}/CaT_{50} metrics for hypokalemia levels between 5.4 mM and 2.9 mM.

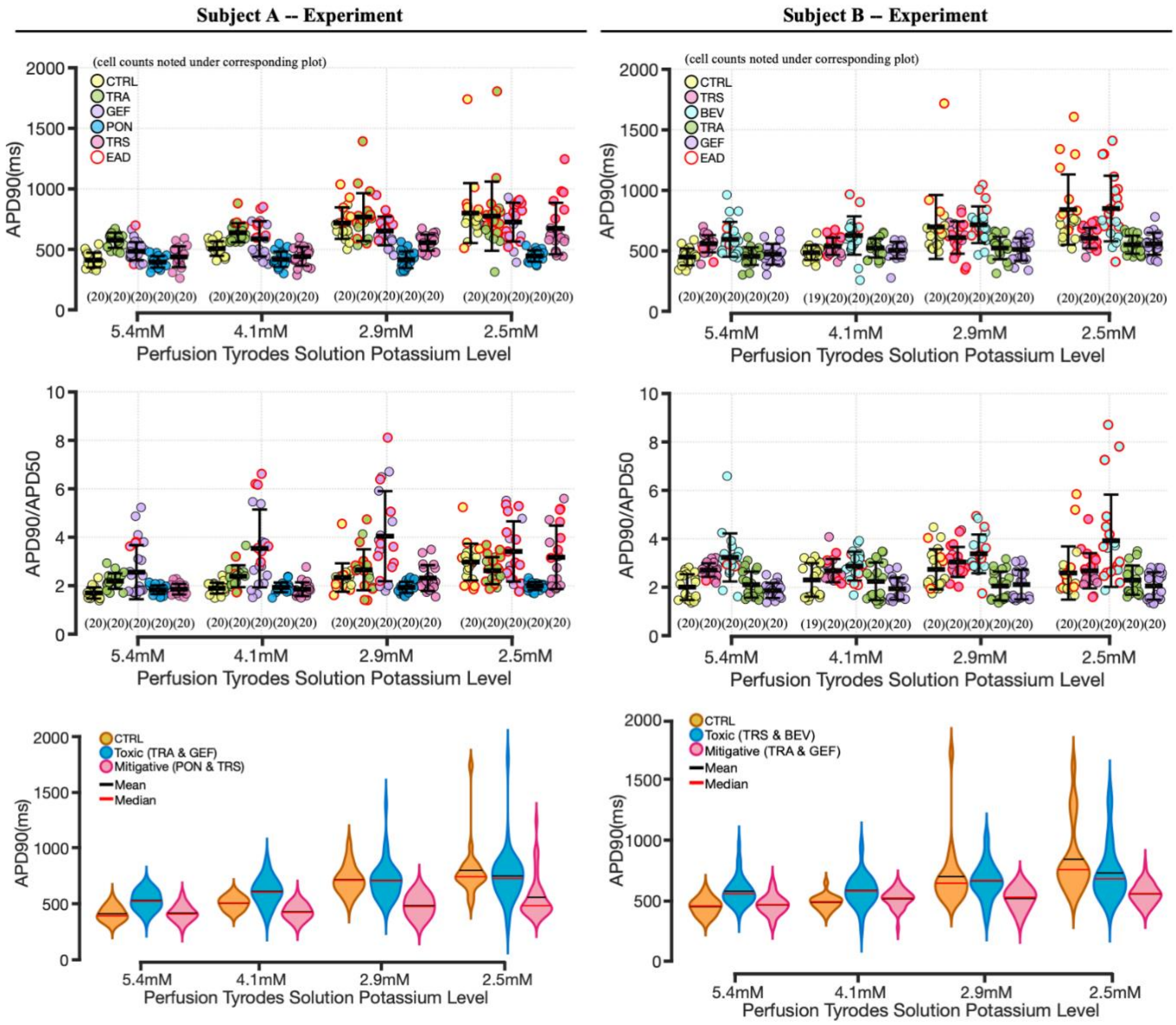

Fig. S10. Hypokalemia predictions and experimental tests: action potential measurements. Experimental results are shown as a function of extracellular [K^+], for 4 drugs, 2 cell lines, and two proarrhythmic markers: APD_{90} and AP triangulation (APD_{90}/APD_{50}). All recorded data points are shown, with superimposed black lines to indicate means ± $\boldsymbol{\sigma}$. In the two bottom panels, the experimental data are grouped as 'toxic' and 'mitigative' based on the prediction result (Figure5). For both subjects, toxic group shows significant increase in both APD_{90} and APD_{90}/APD_{50} metrics for hypokalemia levels between 5.4 mM and 2.9 mM.

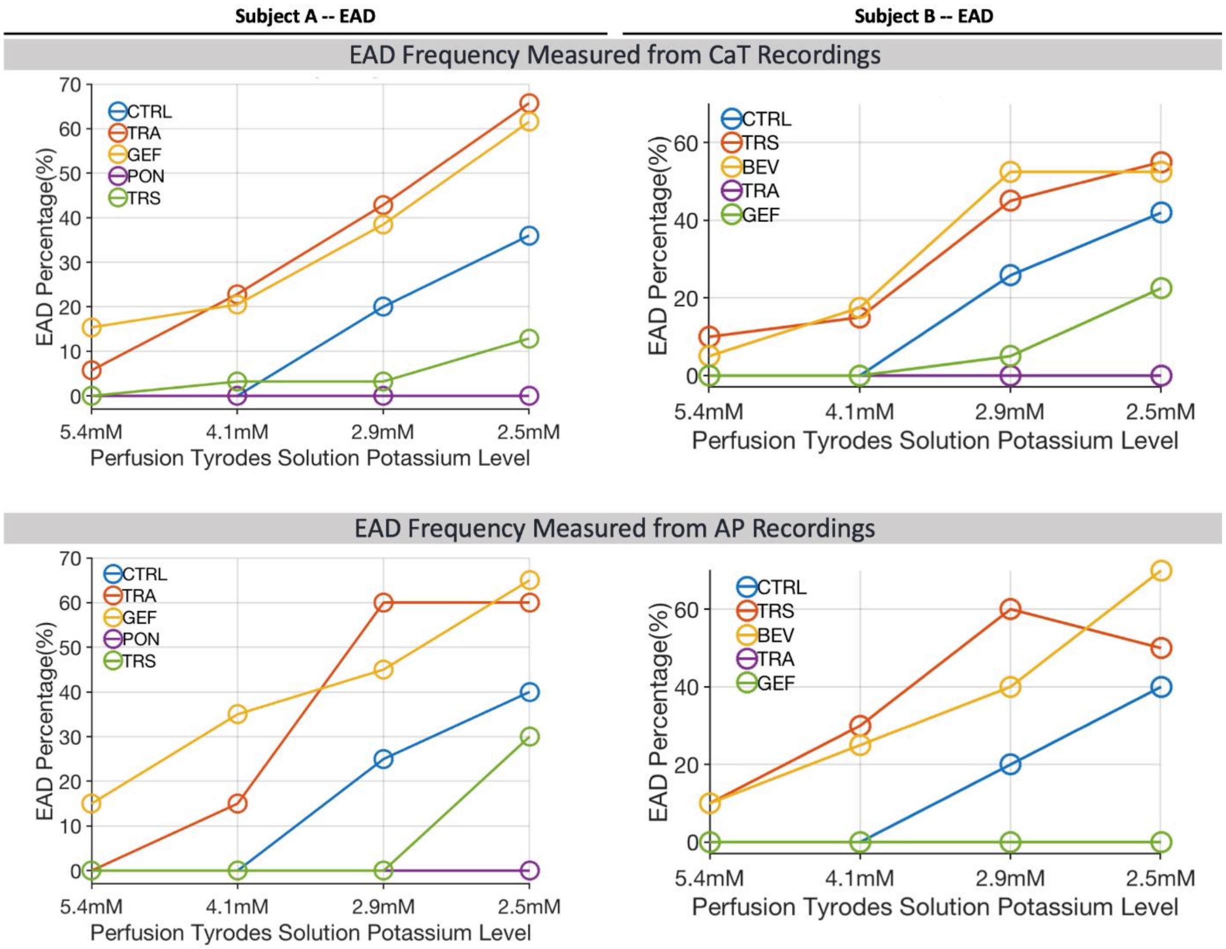

Fig. S11. EAD percentage calculated from either CaT or AP measurements. Percentage of samples exhibiting arrhythmic dynamics, as a function of extracellular [K^+], in the two cell lines (left and right graphs). Arrhythmic dynamics were assessed from recordings of either intracellular [Ca^{2+}] (top graphs) or membrane potential (bottom graphs). Ca^{2+} and membrane potential recordings were made from separate iPSC-CM differentiations, 3-6 months apart, demonstrating reproducibility of the results.

Mechanisms underlying drug and cell line dependence of arrhythmia susceptibility

Figure 6 in the main text uses simulations to explore mechanisms underlying the increase in arrhythmia susceptibility caused by two particular drugs in each of the two cell lines. For trametinib and gefitinib in Cell Line A, and trastuzumab and bevacizumab in Cell Line B, increased susceptibility appears to result from decreased expression of the potassium current I_{K1}. Here these mechanisms are explored in more depth in Supplementary figures, S12 and S13.

Supplementary Figure S12 compares the effects of trametinib and gefitinib between the two cell lines. Because these drugs were predicted to increase susceptibility in Cell Line A but decrease susceptibility in Cell Line B, this presents a helpful comparison. For these simulations, we examined the changes to ionic currents resulting from the two drugs, in the two cell lines, at the level of extracellular [K^+] immediately before arrhythmias occurred (schematic on top of Figure S12). For each ionic current we computed ΔQ, the predicted change in area under the curve. This measure quantifies the change in the total charge carried by the current in drug-treated compared with vehicle-treated cells (schematic on left-hand side of Figure S12; see also Figure S4). The ΔQ values are shown on the right side of Figure S12, with currents grouped into categories.

These results illustrate why trametinib and gefitinib increased arrhythmia susceptibility in Cell Line A but decreased susceptibility in Cell Line B. For I_{K1}, the two drugs are predicted to cause negative ΔQ in both cell lines. This current helps to set the resting potential, so a negative ΔQ will increase susceptibility. However, in Cell Line B only, the two drugs cause a dramatic increase in transient outward K^+ current (I_{to}) due to increased expression of the genes responsible for this current. This positive ΔQ more than compensates for the reduction in I_{K1}, leading to decreased rather than increased susceptibility to hypokalemia, as the experiments confirmed.

Smaller changes to additional currents can also contribute to the increased (Cell Line A) or decreased (Cell Line B) susceptibility that is predicted and observed after treatment with these drugs. The bottom part of the figure summarizes these changes schematically.

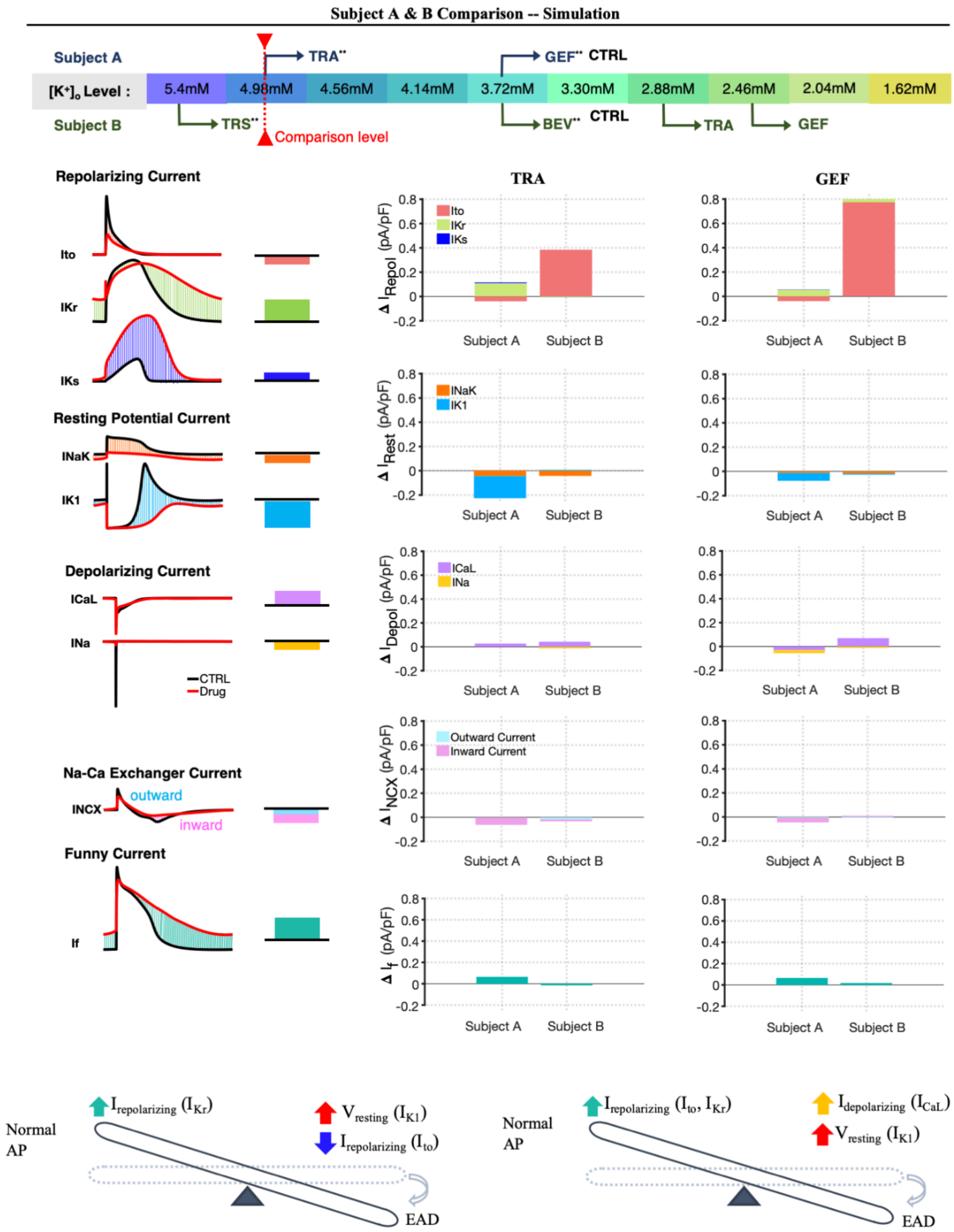

Fig. S12. Mechanisms underlying increased (Cell Line A) or decreased (Cell Line B) arrhythmia susceptibility. See text above for complete explanation of these simulations.

The curious case of trastuzumab in Cell Line B

Simulations of trastuzumab in subject B generated unusual predictions, namely that the arrhythmic waveform at normal [K^+] became non-arrhythmic under mild hypokalemia, then developed EADs with further reductions in extracellular [K^+]. We performed additional analyses to understand the mechanisms underlying this intriguing result. First, we examined the predicted AP waveforms at three levels: (1) the control level of 5.4 mM [K^+], where spontaneous beating was faster than pacing; (2) 4.56 mM [K^+], where spontaneous beating stopped; and (3) 4.14 mM [K^+], where EADs occurred. These cellular behaviors are shown in Supplementary Figure S13A.

To investigate the currents responsible for the spontaneous depolarization at normal [K^+], we examined two time periods that preceded the electrical stimulation, indicted by the pink and green boxes. During the early period indicated by the green box (Figure S13B), we examined average values of the resting potential, I_{Na}, I_f, and I_{K1}. Resting potential was predicted to be higher (less negative), and inward I_{Na} predicted to be larger, in trastuzumab-treated cells. Moreover, these differences were substantial at 5.4 mM [K^+] but less dramatic at 4.56 mM or 4.14 mM [K^+]. This occurred because the membrane potential at 5.4 mM was close to the peak of the I_{Na} "window current" (Figure S13B, left). This window current led to a large peak in I_{Na} and spontaneous depolarization at 5.4 mM [K^+], as shown by the currents in the pink boxes (Figure S13C).

Next, we explored the currents that produced the EAD at 4.14mM [K^+] by computing ΔQ for nine currents during the period before the EAD, as indicated by the blue box. This showed, again, that increased inward I_{Na} contributes to arrhythmia susceptibility by producing the EAD upstroke (Figure S13B).

At 4.56 mM, in contrast, arrhythmias were not observed. This occurred because hypokalemia lowered the resting potential, which pushed cells away from the I_{Na} window current peak and allowed them to evade spontaneous depolarizations. Moreover, at that particular level of [K^+], the insult was not severe enough to cause I_{Na} to induce EADs.

Taken together, these results predict potential mechanisms by which trastuzumab, in Cell Line B, produced unusual, biphasic behavior with changes in extrellular [K^+].

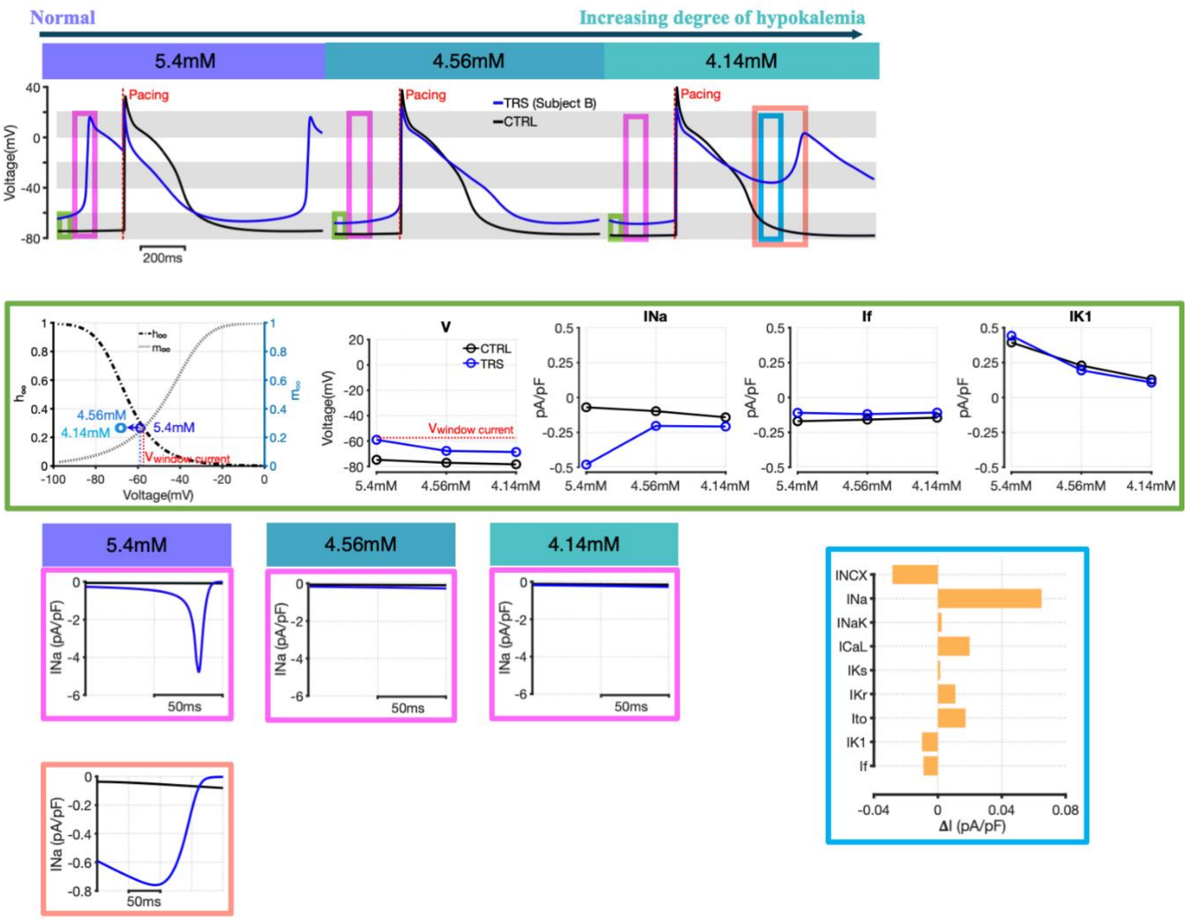

Fig. S13. The curious case of trastuzumab in Cell Line B. See text above for a complete explanation of the results.

SUPPLEMENTARY REFERENCES

1. Schlaeger TM, Daheron L, Brickler TR, Entwisle S, Chan K, Cianci A, DeVine A, Ettenger A, Fitzgerald K, Godfrey M, Gupta D, McPherson J, Malwadkar P, Gupta M, Bell B, Doi A, Jung N, Li X, Lynes MS, Brookes E, Cherry AB, Demirbas D, Tsankov AM, Zon LI, Rubin LL, Feinberg AP, Meissner A, Cowan CA and Daley GQ. A comparison of non-integrating reprogramming methods. *Nat Biotechnol*. 2015;33:58-63.
2. Tohyama S, Hattori F, Sano M, Hishiki T, Nagahata Y, Matsuura T, Hashimoto H, Suzuki T, Yamashita H, Satoh Y, Egashira T, Seki T, Muraoka N, Yamakawa H, Ohgino Y, Tanaka T, Yoichi M, Yuasa S, Murata M, Suematsu M and Fukuda K. Distinct metabolic flow enables large-scale purification of mouse and human pluripotent stem cell-derived cardiomyocytes. *Cell Stem Cell*. 2013;12:127-37.
3. Paci M, Hyttinen J, alto-Setala K and Severi S. Computational models of ventricular- and atrial-like human induced pluripotent stem cell derived cardiomyocytes. *Ann Biomed Eng*. 2013;41:2334-2348.
4. Rice JJ, Wang F, Bers DM and de Tombe PP. Approximate model of cooperative activation and crossbridge cycling in cardiac muscle using ordinary differential equations. *Biophys J*. 2008;95:2368-90.
5. Dubois NC, Craft AM, Sharma P, Elliott DA, Stanley EG, Elefanty AG, Gramolini A and Keller G. SIRPA is a specific cell-surface marker for isolating cardiomyocytes derived from human pluripotent stem cells. *Nat Biotechnol*. 2011;29:1011-8.
6. Kho C, Lee A and Hajjar RJ. Altered sarcoplasmic reticulum calcium cycling--targets for heart failure therapy. *Nat Rev Cardiol*. 2012;9:717-33.
7. Shah RR and Hondeghem LM. Refining detection of drug-induced proarrhythmia: QT interval and TRIaD. *Heart Rhythm*. 2005;2:758-72.
8. Sakmann B and Trube G. Conductance properties of single inwardly rectifying potassium channels in ventricular cells from guinea-pig heart. *J Physiol*. 1984;347:641-57.
